# Supplementary material for: Efficacy of regular gargling with a cetylpyridinium chloride plus zinc containing mouthwash can reduce upper respiratory symptoms
Source: PLoS One. 2025 Feb 26;20(2):e0316807. doi: 10.1371/journal.pone.0316807 (PMC11864509; doi:10.1371/journal.pone.0316807)
Supplement: S1 Appendix — (DOCX) [file pone.0316807.s001.docx]

**UNIVERSIDADE FEDERAL DE PELOTAS**

**FACULDADE DE ODONTOLOGIA**

**DEPARTAMENTO DE SEMIOLOGIA E CLÍNICA**

Projeto de pesquisa:

**EFICÁCIA DO ENXAGUATÓRIO BUCAL DE CLORETO DE CETILPIRIDÍNIO E ZINCO NA DIMINUIÇÃO DA OCORRÊNCIA DE SINTOMAS ASSOCIADOS À TEMPORADA DE GRIPE E RESFRIADO – UM ENSAIO CLÍNICO RANDOMIZADO**

FRANCISCO WILKER MUSTAFA GOMES MUNIZ

Pelotas

Maio de 2022

**EQUIPE DE TRABALHO**

FRANCISCO WILKER MUSTAFA GOMES MUNIZ – Mestre e Doutor em Periodontia. Professor de Periodontia da Universidade Federal de Pelotas (UFPel). Professor do Programa de Pós-Graduação em Odontologia (PPGO) UFPel.

CASSIANO KUCHENBECKER RÖSING – Mestre e Doutor em Periodontia. Professor

titular de Periodontia da UFRGS. Professor permanente do PPG Odontologia UFRGS.

MAÍSA CASARIN – Mestre e Doutora em Periodontia. Professor de Periodontia da UFPel. Professora do PPGO-UFPel.

NATÁLIA MARCUMINI POLA – Mestre e Doutora em Periodontia. Professor de Periodontia da UFPel. Professora do PPGO-UFPel.

TACIANE MENEZES DA SILVEIRA – Mestre em Periodontia. Aluna de doutorado em Clínica Odontológica/Periodontia do PPGO-UFPel.

GUILHERME AZÁRIO DE HOLANDA – Aluno de doutorado em Clínica Odontológica/Periodontia do PPGO-UFPel.

FRANCISCO HECKTHEUER SILVA – Aluno de mestrado em Clínica Odontológica/Periodontia do PPGO-UFPel.

LARISSA VIANA DE OLIVEIRA – Aluna de graduação em Odontologia da UFPel.

PEDRO PAULO DE ALMEIDA DANTAS – Aluno de graduação em Odontologia da UFPel.

**RESUMO**

O objetivo deste estudo será avaliar a eficácia da limpeza regular da orofaringe na incidência de sintomas associados à gripe e resfriado. Um total de 150 indivíduos serão randomizados para um dos dois regimes experimentais e acompanhados pelo período de 90 dias. Os regimes experimentais compreendem: controle, com uso de dentifrício e escova de dentes macia; e teste, com uso de dentifrício, escova de dentes macia e enxaguatório (colutório) bucal para gargarejo contendo cloreto de cetilpiridínio (CPC) (0,075%) e zinco em uma solução livre de álcool. Todos os produtos estão disponíveis comercialmente e serão fornecidos pela equipe de pesquisa. Os participantes serão instruídos a escovar os dentes duas vezes ao dia, por dois minutos. Aqueles que forem alocados para o regime teste, farão gargarejo com 20 ml do colutório após cada escovação. Na visita inicial, os participantes receberão os produtos e instruções para uso, assim como um questionário de registro diário. Será solicitado que os participantes enviem seus registros diários uma vez na semana, por meio de aplicativo de envio de mensagem. A proporção de dias sem nenhum dos sintomas avaliados será utilizada para desfecho principal. Além disso, exames de tecidos moles e duros serão realizados na consulta inicial, após 30 e 90 dias de acompanhamento. Possíveis eventos adversos serão coletados ao longo de todo o estudo. Os grupos serão comparados por meio teste de qui-quadrado e o nível de significância será estabelecido em 95%.

**Palavras-chave:** Influenza, Orofaringe, Cetilpiridínio.

**SUMÁRIO**

[1. INTRODUÇÃO 6](#_Toc102752428)

[2. OBJETIVO 8](#_Toc102752429)

[3. HIPÓTESE 9](#_Toc102752430)

[4. METODOLOGIA 10](#_Toc102752431)

[4.1. Desenho experimental e local de realização 10](#_Toc102752432)

[4.2. População do estudo 10](#_Toc102752433)

[4.2.1. Cálculo amostral 10](#_Toc102752434)

[4.2.2. Critérios de inclusão 10](#_Toc102752435)

[4.2.3. Critérios de exclusão 11](#_Toc102752436)

[4.3. Produtos testes 11](#_Toc102752437)

[4.4. Procedimentos experimentais 12](#_Toc102752438)

[4.4.1. Seleção dos participantes 12](#_Toc102752439)

[4.4.2. Exame de tecidos bucais moles e duros 13](#_Toc102752440)

[4.4.3. Alocação dos participantes em cada grupo 13](#_Toc102752441)

[4.4.4 Produtos de uso domiciliar e instruções de uso aos participantes 14](#_Toc102752442)

[4.4.5 Avaliações subsequentes 14](#_Toc102752443)

[4.5. Acompanhamento e descontinuidade dos participantes 15](#_Toc102752444)

[4.6. Gravidez 15](#_Toc102752445)

[4.7. Análise estatística 16](#_Toc102752446)

[5 CONSIDERAÇÕES ÉTICAS 17](#_Toc102752447)

[5.1. Aprovação pelo Comitê de Ética da UFPel 17](#_Toc102752448)

[5.2. Riscos e benefícios 17](#_Toc102752449)

[5.3. Documentação do estudo 18](#_Toc102752450)

[6 MANEJO DOS PRODUTOS TESTE 20](#_Toc102752451)

[6.1. Produtos testes 20](#_Toc102752452)

[6.2. Armazenagem, manejo e prestação de contas dos produtos testes 20](#_Toc102752453)

[6.3. Administração dos produtos teste 20](#_Toc102752454)

[7. TERAPIA CONCOMITANTE 21](#_Toc102752455)

[8. EVENTOS ADVERSOS 22](#_Toc102752456)

[8.1. Definições 22](#_Toc102752457)

[8.2. Relatórios e documentações de eventos adversos 22](#_Toc102752458)

[8.3. Procedimentos gerais para todos os eventos adversos 22](#_Toc102752459)

[9. NOVOS ACHADOS 24](#_Toc102752460)

[REFERÊNCIAS BIBLIOGRÁFICAS 25](#_Toc102752461)

[CRONOGRAMA 26](#_Toc102752462)

[ORÇAMENTO 28](#_Toc102752463)

[APÊNDICE A - TERMO DE CONSENTIMENTO LIVRE E ESCLARECIDO 30](#_Toc102752464)

[APÊNDICE B. FORMULÁRIO DE REAÇÕES ADVERSAS 36](#_Toc102752465)

[APÊNDICE C - QUESTIONÁRIO DE SAÚDE 38](#_Toc102752466)

[APÊNDICE D. FORMULÁRIO DE EXAMES DE TECIDOS BUCAIS MOLES E DUROS 42](#_Toc102752467)

[APÊNDICE E. FORMULÁRIO DE EXAME INICIAL 43](#_Toc102752468)

[APÊNDICE F. QUESTIONÁRIO DE REGISTRO DIÁRIO 44](#_Toc102752469)

[APÊNDICE G. RÓTULO DE INSTRUÇÕES PARA O PARTICIPANTE 47](#_Toc102752470)

[APÊNDICE H. FORMULÁRIO DE VISITA 48](#_Toc102752471)

# INTRODUÇÃO

As infecções respiratórias de origem viral são responsáveis por altos graus de morbidade e de mortalidade ao redor do mundo. Dentre as infecções respiratórias virais, aquelas causadas pelo vírus Influenza são uma das mais comuns. Além disso, seu impacto sobre os sistemas de saúde é de grande importância (FRASER, TOMBE-MDEWA, KOHLI-LYNCH, *et al*., 2022).

A partir do surgimento da pandemia COVID -19, o interesse por infecções respiratórias virais cresceu, e uma série de estudos foram realizados para compreender o papel de diferentes estratégias que pudessem mitigar os efeitos das infecções respiratórias virais. Tanto para as infecções por influenza quanto para o COVID-19, a vacinação tem-se mostrado uma estratégia preventiva e de limitação de danos com graus satisfatórios de eficiência, sob aspectos individuais e, principalmente, coletivos.

Entretanto, em adição à vacinação, diferentes estratégias têm sido propostas. Especificamente no caso da cavidade bucal, estudos têm sido realizados com diferentes agentes já utilizados anteriormente com objetivos de coadjuvar a higiene bucal foram revisitados em relação à sua capacidade antiviral (FERNANDEZ, GUEDES, LANGA, *et al*., 2022), com vistas a determinar eventuais/potenciais efeitos sobre infecções respiratórias virais, tendo em vista que a orofaringe é um dos espaços afetados e potencial porta de entrada da infeção. Contudo, boa parte dessa literatura advém de estudos *in vitro*, havendo uma lacuna na literatura sobre a eficácia clínica antiviral desses produtos de higiene bucal.

Assim como a lavagem/higiene das mãos tem sido considerada uma medida de impacto na prevenção de disseminação de infecções virais, hipotetiza-se que medidas de higiene bucal e da orofaringe possam, também, apresentar tais efeitos benéficos. Dentro desse contexto, ensaios clínicos, que verifiquem a eficácia clínica desses agentes, são necessários.

Nesse sentido, soluções colutórias têm sido estudadas em relação a seu potencial antiviral. Quando soluções a base de clorexidina são avaliadas, em revisão sistemática da literatura, observa-se que podem apresentar um efeito virucida interessante sobre vírus influenza e mesmo SARS-COV 2 (FERNANDEZ, GUEDES, LANGA, *et al*., 2022).

Outros estudos têm sido realizados e procuraram avaliar os potenciais de soluções usadas localmente na boca/região orofaringe no contexto das infeções virais. Em estudo *in vitro*, usando dois dentifrícios e duas soluções para bochecho disponíveis comercialmente, observou-se que enxagues com peróxido de hidrogênio, ou com cloreto de cetilpiridínio, ou uso de dentifrícios com fluoreto estanoso apresentaram efeitos antivirais importantes (RAMJI, CIRCELLO, WINSTON, *et al.*, 2022).

O cloreto de cetilpiridínio (CPC) é um composto quaternário de amônio, com alta segurança e uso disseminado como coadjuvante a procedimentos de higiene bucal. Apresenta uma boa adesão, por parte dos pacientes, e seu efeito antibacteriano na microbiota bucal é inconteste (LANGA, MUNIZ, COSTA, *et al.*, 2021). Além disso, recentemente, a literatura tem demonstrado que a adição do zinco (Zn), nos colutórios que contêm CPC, têm demonstrado efeitos clínicos superiores, quando comparados com colutórios que contém apenas CPC (RÖSING, CAVAGNI, GAIO *et al*., 2017) ou óleos essenciais (LANGA, CAVAGNI, MUNIZ *et al*., 2021). Contudo, seu potencial antiviral ainda é pouco testado, mas apresenta potencial e tem sido recomendado como bochecho pré-operatório para procedimentos odontológicos (RETAMAL-VALDES, SOARES, STEWART, *et al*., 2017).

Com base nesses potenciais e na necessidade de estudos clínicos, além do eventual efeito do uso tópico de antissépticos orais para diminuir infecções virais, justifica-se este estudo, o qual se pretende avaliar o efeito de uso de solução de CPC+Zn sob a forma de gargarejo na ocorrência de sintomas de gripe e resfriado. A hipótese em estudo é que a presença local de CPC+Zn pode diminuir a ocorrência de sintomas gripais quando comparado com a ausência da presença desses ingredientes ativos.

# OBJETIVO

O objetivo deste estudo será de avaliar a eficácia da limpeza regular da orofaringe, na incidência e duração dos sintomas associados à gripe e resfriado, com o uso de gargarejo de CPC+Zn em comparação com a ausência de gargarejo.

# HIPÓTESE

A hipótese operacional é de que haverá redução significativa dos sintomas associados à gripe e resfriado após a utilização do produto à base de CPC+Zn, comparados ao regime experimental controle.

# METODOLOGIA

# 4.1. Desenho experimental e local de realização

Este é um ensaio clínico randomizado de fase III, controlado, paralelo, de centro único, para avaliar a eficácia da limpeza regular da orofaringe na incidência e duração dos sintomas associados à gripe e resfriado. O estudo será realizado nas clínicas odontológicas da Faculdade de Odontologia (FO) da Universidade Federal de Pelotas (UFPel).

# População do estudo

Cento e cinquenta (150) indivíduos, com idades entre 18-70 anos, serão incluídos nesse estudo.

### Cálculo amostral

O tamanho amostral de 150 indivíduos (75 por grupo) foi baseado em dados de arquivo interno do patrocinador desse estudo. Foi determinado que 60 indivíduos por grupo são necessários para um poder de 80% para a ocorrência de sintomas gripais. Tendo em vista a natureza do estudo e a demanda por respostas diárias a questionário, supõe-se uma taxa de atrição de 25% no decorrer do estudo.

- - 1. Critérios de inclusão

Os indivíduos devem encaixar-se em todos os critérios seguintes:

1. Assinar este termo de consentimento livre e esclarecido (TCLE) (Apêndice A);
2. Homens ou mulheres, com idade entre 18 e 70 anos;
3. Boa saúde geral sistêmica, conforme determinado pelos investigadores do estudo;
4. Disponibilidade de 90 dias para a participação no estudo;
   - 1. Critérios de exclusão

Durante o transcorrer do estudo, os seguintes critérios de exclusão serão considerados:

1. Estar participando de qualquer outro estudo clínico;
2. Estar grávida ou amamentando;
3. Apresentar histórico de alergias aos produtos de higiene bucal, produtos de higiene pessoal, ou a seus ingredientes;
4. Apresentar irritação bucal ou estar fazendo uso de sprays anestésicos orais;
5. Apresentar diabetes;
6. Estar realizando tratamento odontológico extenso ou cirurgia bucal durante o estudo;
7. Apresentar imunocomprometimento (HIV, AIDS, terapia medicamentosa imunossupressora);
8. Fazer uso de prótese total;
9. Apresentar síndrome do túnel carpal ou artrite nas mãos.
10. Participante que, de forma substancial, deixar de seguir os protocolos requeridos;
11. Participante que falhar em comparecer às consultas agendadas;
12. Participante que for tratado, durante o período do estudo, com medicações que possam interferir com os parâmetros em análise do estudo;
13. Participante que for atendido por algum serviço de médico ou odontológico, e que este possa interferir nos parâmetros em análise do estudo;
14. Participante que desenvolver reações adversas sérias. O investigador irá, imediatamente, registrar as informações no Formulário de Reações Adversas (Apêndice B);
15. O participante que opte por encerrar sua participação no estudo;
16. Participante reportar estar grávida no decorrer do estudo.

## **Produtos testes**

Os participantes serão randomizados em um dos seguintes regimes experimentais e receberão os produtos conforme segue:

Grupo Controle:

● Pasta de dentes Colgate máxima proteção anticáries^®^.

● Escova de dentes macia disponível comercialmente.

Grupo Teste:

● Enxaguante bucal contendo 0,075% de CPC e 0,28% de lactato de zinco, em uma base livre de álcool, disponível comercialmente no Brasil.

● Pasta de dentes Colgate máxima proteção anticáries^®^.

- Escova de dentes macia disponível comercialmente.

## **Procedimentos experimentais**

### Seleção dos participantes

Os participantes da pesquisa que assinarem o termo de consentimento livre esclarecido e responderem ao questionário de saúde (Apêndices A e C) receberão um exame clínico intraoral para identificar se preenchem os critérios de inclusão e exclusão. Todos os indivíduos receberão uma avaliação de seus tecidos bucais moles e duros, os achados serão registrados no Formulário de Tecidos Bucais Moles e Duros (Apêndice D). O questionário inicial será registrada no Formulário de Exame Inicial (Apêndice E). Os primeiros 150 indivíduos que preencherem aos critérios de inclusão, assinarem o TCLE e o questionário de saúde serão incluídos no estudo. Após as avaliações iniciais, todos os indivíduos receberão um questionário para o registro diário dos sintomas associados à gripe e resfriado (Apêndice F). Esse formulário será entregue no formato físico, com o questionário para os 90 dias do estudo, para facilitar o preenchimento pelo participante. Uma vez por semana, os participantes deverão enviar fotografias de seus registros diários realizados. Esse envio será realizado por meio de aplicativo de envio de mensagem. Os participantes serão ressarcidos de valores compatíveis com pacotes de internet. A equipe de pesquisa compromete-se em fazer o download dos registros e armazena-los em dispositivos em drives físicos externos, não conectados a internet. As mensagens serão apagadas imediatamente após os downloads. Além disso, o sigilo aos dados dos participantes ficará assegurado durante todo o armazenamento dos dados e demais procedimentos desse estudo.

A seleção dos participantes será do tipo conveniência a partir de convite verbal nas diferentes clínicas da FO-UFPEL. Além disso, o recrutamento poderá contar com postagens em mídias sociais, que explicitem os objetivos principais do presente estudo. Aqueles que expressarem algum interesse em participar do estudo receberão o TCLE em duas vias e, após leitura e assinatura do mesmo, serão avaliados com base nos seguintes exames:

### Exame de tecidos bucais moles e duros

Todos os indivíduos receberão um exame dos tecidos bucais moles e duros. Esse exame será realizado por um único examinador treinado e calibrado. Com o auxílio de um odontoscópio e da iluminação artificial da cadeira odontológico, o exame incluirá uma avaliação do palato duro e mole, mucosa gengival, mucosa bucal, áreas mucogengivais, língua, áreas sublingual e submandibular, glândulas salivares e áreas tonsilares e faríngeas. Os achados serão registrados no Formulário de Exames de Tecidos Moles e Duros (Apêndice D). Caso haja necessidade, os indivíduos serão imediatamente encaminhados ao Serviço de Estomatologia da Faculdade de Odontologia da UFPel.

### 4.4.3. Alocação dos participantes em cada grupo

Os participantes incluídos serão randomicamente alocados para um dos dois grupos do estudo, por meio de um website (randomization.org). O cirurgião-dentista examinador e seu auxiliar não receberão informação, durante todo o estudo, a respeito do grupo de alocação de cada participante. Os produtos serão cobertos por um invólucro no intuito de manter o sigilo de alocação. Os produtos serão distribuídos por um pesquisador não envolvido nas avaliações clínicas, em área separada do local de realização de exame e entrevistas, e colocados em uma sacola lacrada para evitar a percepção de quaisquer diferenças na embalagem e aparência dos produtos utilizados entre os dois grupos experimentais. Informações no rótulo consistirão de um código do grupo de estudo, instruções para o uso domiciliar e informações de segurança, incluindo contatos de emergência (Apêndice G).

Os participantes serão numerados, como forma de identificação, cronologicamente de 001 a 150, à medida que forem incluídos no estudo. Os participantes que residem na mesma casa serão designados para o mesmo grupo experimental, ou seja, receberão os mesmos produtos.

Na visita inicial, como forma de orientação os participantes serão instruídos a praticarem a higiene bucal de acordo com o regime experimental designado por um membro da equipe de pesquisa. No caso do regime controle, os participantes serão solicitados a escovar os dentes, por 2 minutos, com o dentifrício, duas vezes por dia. No regime teste, além da escovação, por dois minutos, com o dentifrício, os participantes deverão gargarejar com a substância oferecida por 30 segundos, duas vezes por dia.

### 4.4.4 Produtos de uso domiciliar e instruções de uso aos participantes

Após a avaliação odontológica, todos os sujeitos irão receber produtos de acordo com o regime designado para uso doméstico:

Grupo controle: receberão uma escova de dentes com cerdas macias para adultos e um dentifrício. Instrução para o uso: escovar os dentes por dois minutos, 2 vezes ao dia (de manhã e à noite) com o dentifrício fornecido.

Grupo teste: receberão uma escova de dente com cerdas macias para adultos, enxaguante bucal e um dentifrício. Instrução para o uso: escovar os dentes por 2 minutos, duas vezes ao dia (de manhã e à noite) com o dentifrício fornecido. Após as escovações, fazer gargarejo com o enxaguante bucal (20 ml) por 30 segundos, duas vezes por dia (de manhã e à noite), e depois cuspir.

Os participantes serão instruídos a realizar os procedimentos descritos acima e usar apenas o produto designado ao seu grupo durante o período do estudo. Os produtos serão fornecidos adicionalmente nas visitas subsequentes. Não haverá restrição quanto aos hábitos alimentares durante o estudo, tampouco a limpeza entre os dentes. Após a conclusão do estudo, os participantes serão instruídos a devolver todos os produtos usados.

### 4.4.5 Avaliações subsequentes

Avaliações subsequentes serão realizadas com 30 e 90 dias de uso dos produtos designados. Os participantes irão comparecer ao local de realização da pesquisa para serem avaliados e receberem produtos adicionais, de acordo com o grupo ao qual foram alocados. Durante estas visitas será realizada uma Avaliação Bucal dos Tecidos Moles e Duros (Apêndice D). Um Formulário de Visita (Apêndice H) será preenchido para todos os participantes do estudo.

Após os 90 dias de experimento, todos os participantes serão instruídos a não utilizarem mais os produtos fornecidos, retornando aos seus hábitos normais de higiene bucal. Todos os produtos não utilizados serão entregues a um assistente não envolvido no exame clínico e serão descartados seguindo as diretrizes de descarte de resíduos da UFPel.

## **Acompanhamento e descontinuidade dos participantes**

Todos os participantes da pesquisa randomizados e que receberem os produtos serão acompanhados de acordo com o seguinte protocolo:

Os participantes serão considerados como tendo completado o estudo se forem reavaliados ao final dos 90 dias. Serão considerados como perda de seguimento somente se nenhum contato tiver sido estabelecido durante a finalização do estudo, havendo, portanto, informações insuficientes para determinar o estado do participante da pesquisa. Esforços serão feitos para determinar as razões pelas quais o participante não retornou para a(s) visita(s) necessária(s) ou os motivos de sua retirada do estudo. Os participantes sairão do estudo se qualquer uma das seguintes situações acontecer:

1. Participante que, de forma substancial, deixar de seguir os protocolos requeridos;
2. Participante que falhar em comparecer às consultas agendadas;
3. Participante que for tratado, durante o período do estudo, com medicações que possam interferir com os parâmetros em análise do estudo;
4. Participante que for atendido por algum serviço de médico ou odontológico, e que este possa interferir nos parâmetros em análise do estudo;
5. Participante que desenvolver reações adversas sérias. O investigador irá, imediatamente, registrar as informações no Formulário de Reações Adversas (Apêndice B);
6. O participante que opte por encerrar sua participação no estudo;
7. Participante reportar estar grávida no decorrer do estudo.

O pesquisador será responsável por notificar prontamente o Comitê de Ética em Pesquisa da UFPel sobre todos os participantes que saírem prematuramente do estudo que apresentarem efeitos adversos graves.

## **Gravidez**

Não há intenção de incluir mulheres grávidas no presente estudo. Caso alguma mulher engravide durante a realização desse estudo clínico, sua participação será encerrada e haverá uma notificação do evento pelo pesquisador responsável.

## **4.7. Análise estatística**

Teste qui-quadrado será realizado para os dados de sexo, e um teste t independente, para a variável idade, afim de testar a hipótese de que os grupos apresentam equilíbrio de distribuição das variáveis. A proporção de dias sem sintomas (ou seja, nenhum dos 10 sintomas estava presente) será usada para a variável de desfecho primário. Teste qui-quadrado será utilizado para comparar a proporção de dias sem sintomas para os grupos experimentais. Análise da variância poderá ser utilizada para comparação entre os grupos. Nível de significância será estabelecido em 95%.

# CONSIDERAÇÕES ÉTICAS

## **5.1. Aprovação pelo Comitê de Ética da UFPel**

O presente projeto será submetido para avaliação do Comitê de Ética em Pesquisa (CEP) da UFPel, via Plataforma Brasil, e só será iniciado após aprovações. O estudo será conduzido em concordância com as normas de “Boas Práticas Clínicas”. Todos os participantes da pesquisa assinarão um TCLE (Apêndice A).

Ressalte-se que o produto a ser testado já possui formulação semelhante em diferentes produtos comercializados no mercado brasileiro. Nesse sentido, é importante pontuar que nenhum dos produtos a serem utilizados no estudo trata-se de um novo produto a ser comercializado.

## **5.2. Riscos e benefícios**

Em geral, não se espera que haja nenhum efeito secundário do uso de nenhum dos produtos em teste, tendo em vista que são produtos de uso rotineiro e comercialmente disponíveis. Entretanto, existe a possibilidade de irritação de tecidos moles ou sensibilidade dentinária temporária com o uso desses produtos, como com qualquer produto de higiene bucal que regularmente se utiliza. Ao ocorrerem essas condições, é reconhecido que as mesmas desapareçam com a suspensão do uso do produto. O exame dos tecidos bucais, que será realizado, é utilizado como parte dos cuidados de saúde bucal de rotina. Existe ainda risco de desconforto ao responder às perguntas do questionário. Os participantes necessitarão despender tempo para deslocamento e participação por todo o período do estudo, além da necessidade de aquisição de pacote de dados móveis para os enviados semanais dos questionários aplicados. Por conta disso, os pacientes serão compensados com o valor de R$50,00 (cinquenta reais), a cada visita realizada, com o intuito de compensar as despesas inerentes aos seus deslocamentos à Faculdade de Odontologia da UFPEL. Além disso, a aquisição do pacote de dados móveis também será ressarcida com o valor de R$100,00 (cem reais) durante o último retorno dos participantes.

Não são esperados benefícios diretos na participação deste estudo. Os resultados do estudo podem auxiliar a encontrar métodos alternativos para a diminuição da ocorrência de sintomas gripais e de resfriados. Além disso, ajudar a garantir que o produto seja seguro para a comercialização para o público em geral. A equipe de pesquisa será a responsável pelo atendimento aos eventos adversos oriundos da participação no estudo.

## 5.3. **Documentação do estudo**

Todos os dados relevantes para as avaliações delineadas nesse protocolo devem ser registrados nos formulários fornecidos. Para cada participante será gerado um código ao início do estudo e esse será utilizado em todos os demais formulários durante todo o período experimental, com vistas a garantir anonimato. Ao completar o formulário, o investigador ou auxiliar deverá assinar o documento para aceitar as responsabilidades para fins de registro de dados em cada página do formulário.

Os seguintes formulários de registro dos participantes serão completados pelo pesquisador do estudo de acordo com a seguinte sequência:

**Visita inicial**

●TCLE (Apêndice A)

●Questionário de Saúde (Apêndice C)

●Formulário de Exame Inicial (Apêndice E)

**AVALIAÇÕES CLÍNICAS**

**●**Exame dos Tecidos Bucais Moles e Duros (Apêndice D)

**Visita 2: dia 30**

●Formulário de Visita (Apêndice H)

**AVALIAÇÕES CLÍNICAS**

●Exame dos Tecidos Bucais Moles e Duros (Apêndice D)

**Visita 3: Dia 90**

●Formulário de Visita (Apêndice H)

● Entrega do conjunto de questionários físicos respondidos durante o estudo (Apêndice F)

**AVALIAÇÕES CLÍNICAS**

●Exame dos Tecidos Bucais Moles e Duros (Apêndice D)

# MANEJO DOS PRODUTOS TESTE

## **6.1. Produtos testes**

Todos os produtos testes serão fornecidos pela Colgate-Palmolive e serão manejados de acordo com as normas de biossegurança vigentes na FO-UFPel.

## **Armazenagem, manejo e prestação de contas dos produtos testes**

Os produtos testes serão armazenados em uma área segura, com limite de acesso e em temperatura ambiente. O investigador será diretamente responsável pela acomodação de todos os produtos utilizados ou não pelo estudo. O examinador e auxiliar permanecerão cegos ao tratamento empregado para cada participante. Os registros serão mantidos para documentar a receita e disposição de todos os produtos empregados no estudo, fornecidos pelo investigador para o pesquisador.

## **Administração dos produtos teste**

Todos os participantes receberão os seus produtos teste designados na consulta inicial/inclusão. Os participantes receberão instruções escritas sobre a utilização dos produtos.

●Dosagem do produto teste: Todos os tratamentos serão distribuídos pelo coordenador do estudo no local de realização do estudo.

●Tempo e local de dispensação dos produtos: será realizada no dia/hora de inclusão de cada participante na clínica odontológica.

●Estocagem dos agentes entre consultas: Todos os produtos serão mantidos em área clínica fechada.

# TERAPIA CONCOMITANTE

Se algum participante realizar terapia medicamentosa concomitante, como uma necessidade para o tratamento de alguma condição médica, então**,** a manutenção do participante durante o período experimental do estudo será avaliado e definido a critério do investigador. Entretanto, é de responsabilidade do investigador desqualificar a entrada de qualquer indivíduo que, durante as avaliações iniciais, estiverem utilizando ou consumindo produtos que possam dificultar a interpretação dos resultados do estudo. Todas as medicações utilizadas pelos participantes, no dia de sua inclusão, ou qualquer tempo durante o período do estudo, serão registradas no Formulário de Visita (Apêndice H). Os participantes podem receber medicações para tratar efeitos adversos, sempre que necessário, pelo investigador ou pelo médico do participante.

# EVENTOS ADVERSOS

## **Definições**

Eventos adversos (EA) e eventos adversos sérios (EAS) são definidos pelas Diretrizes de Boas Práticas Clínicas ICH (ICH CGP) da seguinte maneira:

**Eventos adversos:** Qualquer ocorrência médica não convencional, em um paciente ou em participantes de investigação clínica que estão recebendo administração de algum agente farmacológico e que não necessariamente necessita ter uma relação causal com o tratamento. O evento adverso pode, portanto, ser qualquer sinal desfavorável e não intencional (incluindo achados laboratoriais anormais), sintomas ou doenças temporárias associadas com o uso de produtos medicinais, que estejam ou não relacionados com o produto medicinal.

EAs incluem qualquer sinal de deterioração do estado médico do participante, após ser incluído em um estudo. O EA pode envolver qualquer órgão ou sistema que possa ser representado pela nova ocorrência ou deterioração da doença, uma síndrome, um sintoma, um sinal físico, assim como por achados e resultados de exames instrumentais e testes laboratoriais. Qualquer mudança inconveniente desde a inclusão do indivíduo, ocorrendo após a primeira administração do produto-teste do estudo, é considerada um efeito adverso. Todas essas ocorrências devem ser registradas e reportadas apropriadamente, mesmo que elas estejam relacionadas com a medicação do estudo ou não.

## **Relatórios e documentações de eventos adversos**

Todos os formulários de eventos adversos estão fornecidos no Apêndice B.

## **Procedimentos gerais para todos os eventos adversos**

Todas as queixas, sintomas ou sinais que se encaixarem com as definições de efeito adverso deverão ser registradas no Formulário de Reações Adversos (Apêndice B), incluindo o que segue:

- Descrição do evento adverso
- Data do início da reação
- Data da resolução
- Desfecho
- Severidade
- Seriedade
- Relação com a droga do estudo (causalidade)
- Ações realizadas

Eventos adversos serão acessados pela equipe de pesquisa em termos de severidade, relação com o produto em estudo, possíveis etiologias. O formulário de relato de caso propõe que todos os desfechos de eventos adversos registrados sejam reportados em até duas semanas após o fim do estudo. O investigador é responsável por acompanhar todos os efeitos adversos até a resolução ou até que não haja mais nenhuma preocupação clínica, fornecendo esses dados ao financiador. O investigador também é responsável por reportar todos os eventos adversos para o CEP- UFPel, de acordo com as regras e procedimentos estabelecidos pela resolução 466/12.

# NOVOS ACHADOS

Os participantes serão informados de quaisquer novos achados significativos relacionados a produtos ou procedimentos de estudo quando forem conhecidos durante o curso deste estudo clínico. Tais informações podem afetar a decisão do sujeito para continuar a participação no estudo.

# REFERÊNCIAS BIBLIOGRÁFICAS

FRASER, H.; TOMBE-MDEWA, W.; KOHLI-LYNCH, C.; HOFMAN, K.*et al.* Costs of seasonal influenza vaccination in South Africa. **Influenza Other Respir Viruses**, Mar 30 2022.

FERNANDEZ, M. D. S.; GUEDES, M. I. F.; LANGA, G. P. J.; RÖSING, C. K.*et al.* Virucidal efficacy of chlorhexidine: a systematic review. **Odontology**, 110, n. 2, p. 376-392, Apr 2022.

RAMJI, N.; CIRCELLO, B.; WINSTON, J. L.; BIESBROCK, A. R. Virucidal Activity of Over-the-Counter Oral Care Products Against SARS-CoV-2. **Oral Health Prev Dent**, 20, n. 1, p. 185-192, Apr 27 2022.

LANGA, G.P.J.; CAVAGNI, J.; MUNIZ, F.W.M.G.; OBALLE, H.J.R., *et al.* Antiplaque and antigingivitis efficacy of cetylpyridinium chloride with zinc lactate compared with essential oil mouthrinses: Randomized clinical trial. **J Am Dent Assoc**, 152, p. 105-114, Feb 2021.

LANGA, G. P. J.; MUNIZ, F.; COSTA, R.; DA SILVEIRA, T. M.*et al.* The effect of cetylpyridinium chloride mouthrinse as adjunct to toothbrushing compared to placebo on interproximal plaque and gingival inflammation - a systematic review with meta-analyses. **Clin Oral Investig**, 25, n. 2, p. 745-757, Feb 2021.

RETAMAL-VALDES, B.; SOARES, G. M.; STEWART, B.; FIGUEIREDO, L. C.*et al.* Effectiveness of a pre-procedural mouthwash in reducing bacteria in dental aerosols: randomized clinical trial. **Braz Oral Res**, 31, p. e21, Mar 30 2017.

RÖSING, C.K.; CAVAGNI, J.; GAIO, E.J.; MUNIZ, F.W.M.G.; *et al.* Efficacy of two mouthwashes with cetylpyridinium chloride: a controlled randomized clinical trial. **Braz Oral Res**, 31, p. e47, Jul 2017.

# CRONOGRAMA

A previsão de início do presente projeto de pesquisa é para julho de 2022. Contudo, esse projeto terá início somente após aprovação pela Comitê de Ética em Pesquisa da Faculdade de Odontologia da UFPel.

| **Atividade/Mês**  **(após a aprovação do CEP/UFPel)** | **Mês 1** | **Mês 2** | **Mês 3** | **Mês 4** | **Mês 5** | **Mês 6** | **Mês 7** | **Meses**  **8 - 12** |
| --- | --- | --- | --- | --- | --- | --- | --- | --- |
| Revisão de literatura | **X** | **X** | **X** | **X** | **X** | **X** | **X** |  |
| Treinamento do examinador | **X** |  |  |  |  |  |  |  |
| Seleção dos participantes de pesquisa e coleta de dados | **X** | **X** | **X** | **X** | **X** |  |  |  |

| Tabulação e análises dos dados |  |  |  |  | **X** | **X** | **X** |  |
| --- | --- | --- | --- | --- | --- | --- | --- | --- |
| Redação dos artigos, elaboração do relatório de pesquisa e encaminhamento para publicação |  |  |  |  |  |  | **X** | **X** |

# ORÇAMENTO

O presente estudo conta com financiamento completo a ser efetuado Associação Latino Americana para Promoção da Saúde Oral e Pesquisa Odontológica – LAOHA.

| **Produto** | **Preço unitário (R$)** | **Quantidade** | **Total (R$)** |
| --- | --- | --- | --- |
| Custeio de transporte | 50,00 | 450 | 22.500,00 |
| Ressarcimento plano internet celular | 100,00 | 150 | 15.000,00 |
| Caderno – questionário diário | 30,00 | 150 | 4.500,00 |
| Xerox dos questionários | 0,10 | 3.375 unidades | 337,50 |
| Xerox dos termos de consentimento | 0,10 | 400 unidades | 40,00 |
| Impressões | 0,10 | 1000 unidades | 100,00 |
| Dentifrício (regime controle) | 10,00 | 40,5kg | 4.500,00 |
| Dentifrício (regime teste) | 10,00 | 40,5kg | 4.500,00 |
| Escova de dentes multicerdas macia | 5,00 | 400 unidades | 2.000,00 |
| Enxaguante bucal (CPC+Zn) | 15,00 | 450 unidades | 6.750,00 |
| Trio clínico (espelho, pinça clínica, sonda periodontal milimetrada) | 70,00 | 30 unidades | 2.100,00 |
| Equipamentos de proteção individual (luvas, máscara e gorro) | - | - | 500,00 |
| Material de escritório (canetas, lápis, borracha) tinta para impressora (impressora já existente) | - | - | 380,00 |
|  | | **Total** | **63.207,50** |

# APÊNDICE A - TERMO DE CONSENTIMENTO LIVRE E ESCLARECIDO

Prezado(a) Colaborador(a), você está sendo convidado(a) a participar do seguinte estudo:

**. Título da pesquisa: Eficácia do enxaguatório bucal de cloreto de cetilpiridínio e zinco na diminuição da ocorrência de sintomas associados à temporada de gripe e resfriado – um ensaio clínico randomizado**

**. Pesquisador responsável: Prof. Dr. Francisco Wilker Mustafa Gomes Muniz**

**. Instituição a que pertence o pesquisador responsável: Universidade Federal de Pelotas (UFPel)**

**. Local de realização do estudo/coleta de dados: Faculdade de Odontologia – UFPel**

Caro participante,

Você está sendo convidado a participar de um estudo clínico com duração de 90 (noventa) dias. Este consentimento informado objetiva dar-lhe informação que necessitará para decidir se participa do estudo. Por favor, leia este termo de consentimento cuidadosamente. Você pode perguntar sobre o objetivo desta pesquisa, o que será solicitado que você faça, os possíveis riscos e benefícios, seus direitos como participante, e qualquer outra pergunta sobre a pesquisa ou o consentimento que não esteja clara. Após respondermos todas as suas perguntas, você pode decidir se quer participar ou não do estudo.

**1. OBJETIVO DA PESQUISA:** O objetivo deste estudo é avaliar a eficácia de produtos de higiene bucal comercialmente disponíveis na ocorrência de sintomas associados a gripes e resfriados. Com os resultados deste estudo, há a possibilidade de encontrar métodos que possam auxiliar na diminuição da ocorrência de sintomas associados a gripe e resfriado. Este projeto foi avaliado pelo Comitê de Ética em Pesquisa da UFPel, órgão colegiado, de caráter consultivo, deliberativo e educativo, cuja finalidade é avaliar – emitir parecer e acompanhar os projetos de pesquisa envolvendo seres humanos, em seus aspectos éticos e metodológicos, realizados no âmbito da instituição.

**2. PARTICIPAÇÃO NA PESQUISA**: Sua participação na pesquisa se dará por um total de 3 (três) visitas ao local do estudo, ao longo dos 90 (noventa) dias de duração. Se você apresentar os critérios para participação na visita inicial (visita 1), você receberá os produtos de higiene bucal atribuídos.

Ao início do estudo (visita 1), você comparecerá à clínica onde o pesquisador do estudo realizará uma triagem inicial para determinar se você está qualificado para participar do estudo. Você será designado aleatoriamente para um dos dois regimes do estudo: uso de escova de dentes macia + pasta de dentes OU uso de escova macia + pasta de dentes + enxaguatório bucal para gargarejar. Os produtos serão dados para usá-los em casa. Suas informações (idade, data de nascimento, sexo, iniciais do seu nome, etc.) serão coletadas. Você completará um questionário de saúde sobre seu histórico médico, odontológico, condições de saúde bucal e medicações atuais. Um exame de tecidos bucais será realizado para verificar a presença de anormalidades. Você receberá um questionário para preencher em casa diariamente, onde registrará seus sintomas associados à gripe e resfriado. Uma vez por semana (todas as segundas-feiras), você deverá enviar fotos via Whatsapp para o telefone dos questionários da semana anterior.

Você será instruído sobre os requisitos do tratamento deste estudo. Será solicitado que escove seus dentes com a escova e pasta fornecidas duas vezes ao dia, de manhã e à noite, por 2 (dois) minutos. Caso também receba um enxaguante bucal, será solicitado que, após a escovação dos dentes, realize gargarejo com 20 mL (vinte) deste produto, por 30 (trinta) segundos. Ao final você deve cuspir o produto. O uso desse produto, enxaguante bucal, será realizado duas vezes ao dia, sempre após a escovação. Os produtos deverão ser utilizados em todo o período do estudo, 90 (noventa) dias, e nenhum outro produto poderá ser usado. O princípio ativo a ser testado nessa pesquisa já é utilizado em outros produtos.

Gostaríamos de esclarecer que sua participação é totalmente voluntária, tendo você a liberdade de recusar-se a participar, ou mesmo desistir a qualquer momento, e exigir a retirada de sua participação da pesquisa sem que isto acarrete qualquer ônus ou prejuízo à sua pessoa.

Lembre-se: durante este estudo de pesquisa, você não poderá usar outros produtos odontológicos além dos fornecidos (incluindo pastas e escovas de dentes, enxaguante bucal, mascadores de hálito, etc.), ou ter seus dentes limpos ou branqueados em um consultório odontológico. Você deve usar apenas os produtos de teste dados a você durante todo este estudo. Não há restrição quanto a dieta no decorrer do estudo. Tratamentos dentários de rotina não devem ser feitos, entretanto emergências podem ser realizadas. Por favor, informe ao dentista examinador se recebeu tratamento odontológico de emergência, tomou novos medicamentos, recebeu tratamento dental, se ficou grávida ou amamentou.

**3. LOCAL DA PESQUISA:** Será necessário que você compareça à Faculdade de Odontologia da UFPel (Rua Gonçalves Chaves, 457 – Centro, Pelotas, RS) para a realização das três consultas odontológicas, conforme previamente reportado, o que pode levar aproximadamente 20 minutos.

**4. RISCOS E DESCONFORTOS**: Em geral, não se espera que haja nenhum efeito secundário do uso de nenhum dos produtos em teste. Entretanto, existe a possibilidade de ocorrer irritação de tecidos moles ou hipersensibilidade temporária com o uso desses produtos, como com qualquer produto de higiene bucal que você regularmente utiliza. Essas reações não são prejudiciais e desaparecem quando o tratamento é interrompido. Ao ocorrerem essas condições, suspenda o uso dos produtos e contate o pesquisador para orientações. O exame dos tecidos bucais que será realizado é utilizado como parte dos cuidados de saúde bucal de rotina. Existe ainda risco de desconforto ao responder às perguntas do questionário. Você necessitará despender tempo para deslocamento e participação por todo o período do estudo.

Lembre-se: Todos os produtos que você receber serão apenas para o seu uso. Mantenha todos os produtos fora do alcance de crianças e pessoas incapazes de ler ou entender os rótulos, bem como animais de estimação.

Se você apresentar qualquer problema, deverá comunicar-se com o Dr. Francisco Wilker Mustafa Gomes Muniz, no telefone fornecido na consulta clínica. Se não conseguir comunicar-se, por favor, procure seu médico. Qualquer despesa resultante de problema decorrente do uso dos produtos em teste será coberta pelos investigadores.

Se você sofrer algum dano decorrente da participação no estudo, tem direito a assistência integral, imediata e gratuita (responsabilidade dos pesquisadores) e também tem direito a buscar indenização, caso sinta que houve qualquer tipo de abuso por parte dos pesquisadores.

**5. BENEFÍCIOS**: A participação nesse estudo pode não o beneficiar diretamente. Os resultados do estudo, entretanto, podem ajudar a encontrar métodos alternativos que possam auxiliar na diminuição da ocorrência de sintomas de gripe e resfriado da população. Os resultados do estudo também podem ajudar a garantir que o produto seja seguro para a comercialização para o público em geral.

**6. CONFIDENCIALIDADE**: Todas as informações que o(a) Sr.(a) nos fornecer ou que sejam conseguidas por entrevistas e exame clínico odontológico serão utilizadas somente para essa pesquisa. Suas respostas, dados pessoais e avaliações odontológicas e dos sintomas gripais ficarão em segredo e o seu nome não aparecerá em lugar nenhum dos questionários utilizados. Quando os resultados da pesquisa forem divulgados, isto ocorrerá sob forma codificada, para preservar seu nome e manter sua confidencialidade.

Os dados de sua participação neste estudo são confidenciais. Seus registros médico e odontológico serão mantidos de acordo com a Resolução 466/12, que garante a privacidade e sigilo de seus dados. Ainda, a assinatura deste termo não exclui a possibilidade de busca por indenização diante de eventuais danos decorrentes da participação nesta pesquisa.

Os registros deste estudo serão armazenados pelo período de 5 (cinco) anos, em local devidamente restrito, para o qual apenas o pesquisador principal terá acesso em um computador protegido por senha.

Os resultados deste estudo podem ser publicados em uma revista científica, mas seu nome não será revelado.

Sua assinatura abaixo significa que você entende e concorda com as informações acima, e afirma que você se voluntaria para participar. Além disso, você afirma ter respondido com verdade, que lhe foi dada a oportunidade de fazer perguntas sobre o estudo, e que recebeu uma via deste termo de consentimento.

**7. DESPESAS/RESSARCIMENTO:** Os custos do projeto são de responsabilidade do pesquisador. Você será ressarcido em cinquenta reais (R$50,00) em cada uma das três visitas do estudo, para fins de cobrir despesas com transporte e alimentação. Além disso, em sua terceira visita, você ressarcido em cem reais (R$100,00) para cobrir os seus gastos com o plano de internet ao longo de todo o período do estudo.

**8.** **MATERIAIS:** Depois de 30 (trinta) e 90 (noventa) dias de uso dos produtos, você comparecerá à clínica odontológica. Nestas duas visitas, a equipe de pesquisa irá atualizar seu histórico médico/odontológico para verificar qualquer alteração em sua saúde, ou novos medicamentos desde a consulta anterior do estudo. Será feita pergunta para saber se utilizou os produtos da maneira correta e eventuais problemas quanto ao uso dos produtos. Os tecidos moles bucais também serão examinados nestas duas visitas. Será verificado o preenchimento do questionário diário que você recebeu.

Ao final dos 90 dias de duração do estudo, você irá interromper o uso dos produtos designados, retornando a sua rotina de higiene bucal. Todos os produtos que sobrarem devem ser devolvidos na visita final (dia 90). Esses produtos não podem ser compartilhados com ninguém de sua família.

Caso você tenha mais dúvidas ou necessite de maiores esclarecimentos, pode nos contatar nos endereços a seguir ou procurar o Comitê de Ética em Pesquisa da Faculdade de Odontologia da UFPel, cujo endereço consta deste documento.

O Comitê de Ética, de acordo com a Resolução 466/2012-CNS-MS, é um colegiado interdisciplinar e independente, de caráter consultivo, deliberativo e educativo, criado para defender os interesses de participantes de pesquisa em sua integridade e dignidade e para contribuir no desenvolvimento da pesquisa dentro dos padrões éticos. Para garantir os padrões éticos da pesquisa, os tópicos anteriores concedem requisitos mínimos para manter sua integridade e dignidade na pesquisa.

Como segurança jurídica, este termo deverá ser preenchido em **duas vias** de igual teor, sendo uma delas, devidamente preenchida e assinada entregue a você. Além da **assinatura** nos campos específicos pelo pesquisador e por você, solicitamos que sejam **rubricadas todas as folhas** deste documento. Isto deve ser feito por ambos (pelo pesquisador e por você, como participante da pesquisa) de tal forma a garantir o acesso ao documento completo.

Você poderá acionar o/a pesquisador/a responsável ou o Comitê de Ética em Pesquisa, através das informações, endereços e telefones contidos abaixo.

Eu, __________________________________________________________________ declaro que fui devidamente esclarecido e concordo em participar VOLUNTARIAMENTE da pesquisa coordenada pelo **Prof. Dr. Francisco Wilker Mustafa Gomes Muniz**

_____________________________________________ Data: _______________

Assinatura ou impressão datiloscópica do colaborador

Eu, Francisco Wilker Mustafa Gomes Muniz, declaro que forneci todas as informações referentes ao projeto de pesquisa supra-nominado.

________________________________________ Data: _______________

Assinatura do pesquisador

- Qualquer dúvida com relação à pesquisa poderá ser esclarecida com o **pesquisador,** conforme dados e endereço abaixo:

Nome: Francisco Wilker Mustafa Gomes Muniz

Endereço: Rua Gonçalves Chaves, 457

E-mail: wilkermustafa@gmail.com

- Qualquer dúvida com relação aos aspectos éticos da pesquisa poderá ser esclarecida

com o Comitê de Ética em Pesquisa da Faculdade de Odontologia da UFPel, no endereço abaixo:

**Comitê de Ética em Pesquisa da Faculdade de Odontologia da Universidade Federal de Pelotas**

Telefone 53 3260 2801

e-mail cepodonto@ufpel.edu.br

Endereço: Rua Gonçalves Chaves, 457, Centro, Pelotas, RS

HORÁRIO DE FUNCIONAMENTO:
Segunda a Sexta, das 8h às 11h30m e das 14h às 17h30m

#

# APÊNDICE B. FORMULÁRIO DE REAÇÕES ADVERSAS

**Informações sobre o participante:**

| Número do  participante | Iniciais do  participante | Gênero | Idade | Peso | Grupo étnico |
| --- | --- | --- | --- | --- | --- |
|  |  |  |  |  |  |

Em caso de participante mulher, está grávida? ( ) Sim. ( ) Não

Se sim, quantos meses? _________

Data da percepção da reação adversa: ______/______/________

Data do relato da reação adversa: ______/______/_________

Fase do estudo onde o primeiro evento aconteceu: ___________________________________

**Informações sobre o produto utilizado:**

| Nome do produto (número de identificação): |  |
| --- | --- |
| Data de início do uso: | Data de interrupção do uso: |
| Dose: | Frequência diária: |
| Grupo experimental: |  |

**Informações sobre a reação adversa:**

| **Data de início** | **Duração ou data do término** | **Severidade (leve, moderada, severa)** | **Relação com o produto (possível relação; não relacionado; desconhecido)** |
| --- | --- | --- | --- |
|  |  |  |  |

**Descreve a reação adversa em detalhe:** ____________________________________________________________________________________________________________________________________________________________________________________________________________________________________________________________________________________________________________

**Sobre a reação adversa, responda:**

( ) Resolvido – Data:____/____/______

( ) Processo de resolução

( ) Desconhecido/Perda de seguimento

( ) Não resolvido

( ) Resolvido com sequela

( ) Outro: ________________________

**Ação realizada com o produto teste:**

( ) Continuou a utilização

( ) Interrompeu a utilização

( ) Interrompeu a utilização de forma temporária

( ) Reduziu a utilização, especifique:______________________________________________

( ) Desconhecido

( ) Outra

**A reação adversa diminuiu após a interrupção ou redução da dose do produto?**

( ) Sim ( ) Não

**A reação adversa reapareceu após a reintrodução do uso do produto?**

( ) Sim ( ) Não

**Estado do protocolo do participante:**

( ) Protocolo contínuo

( ) Protocolo descontínuo

**Tratamento para a reação adversa/reação adversa séria:** ______________________________________________________________________________________________________________________________________________________

**Dados relevantes do histórico médico:**

( ) Sim, liste-os ( ) Nenhum ( ) Não fornecido ( ) Desconhecido

- Histórico médico, com data de início, se conhecido for: ___________________________________________________________________________

**Medicações concomitantes relevantes:**

( ) Sim, liste-os ( ) Nenhum ( ) Não fornecido ( ) Desconhecido

- Nome da medicação, dose, frequência, datas de início e interrupção da medicação ou

duração da terapia se conhecido for: ___________________________________________________________________________

**Dados laboratoriais relevantes:**

( ) Sim, liste-os ( ) Nenhum ( ) Não fornecido ( ) Desconhecido

- Teste laboratorial, resultados e datas se conhecido for: ___________________________________________________________________________

**Data:______/______/_________**

**Assinatura do dentista examinador: ____________________________________________**

# APÊNDICE C - QUESTIONÁRIO DE SAÚDE

**Data: ____/____/_______ Número do Participante:** ___________________

**Data de nascimento: ____/____/_______** Raça: ______________________

**Telefone para contato:**

**Fumante:** ( ) Sim ( ) Não ( ) Ex-fumante

**HISTÓRICO MÉDICO**

**Nome do médico: ____________________________________________________________**

**Telefone do médico: _________________________________________________________**

**Data da última visita ao médico:** **_______________________________________________**

**Nome de um contato de emergência: ___________________________________________**

**Telefone do contato de emergência: ____________________________________________**

**Sua atual condição de saúde sistêmica é:** ( ) Boa ( ) Regular ( ) Ruim

**Você já foi submetido a algum procedimento cirúrgico sério?** ( ) Sim ( ) Não

**Em caso afirmativo, explique: _________________________________________________**

**Você está fazendo algum tratamento médico?** ( ) Sim ( ) Não

Em caso afirmativo, explique: __________________________________________________

**Este ano (2022), você foi vacinado contra gripe comum (Influenza)?** ( ) Sim ( ) Não

**Você foi vacinado contra SARS-CoV-2 (Covid-19)?** ( ) Sim ( ) Não

**Você já foi tratado ou diagnosticado com alguma dessas condições?**

( ) Sangramento anormal

( ) Hemofilia

( ) Transfusão sanguínea

( ) Úlcera/Colite

( ) Problemas cardíacos

( ) Epilepsia/Desmaios

( ) Asma

( ) Artrite

( ) Glaucoma

( ) Abuso de drogas

( ) Problemas de pele

( ) Doenças do sangue

( ) Quimioterapia/Radioterapia

( ) Diabetes/Açúcar no sangue anormal

( ) Dificuldades respiratórias

( ) Problemas pulmonares

( ) Pressão sanguínea alta ou baixa

( ) Anemia

( ) AIDS

( ) Câncer/Tumor

( ) Doença nos rins ou fígado

( ) Hepatite/Icterícia

( ) Enfisema

( ) Febre reumática

**Por favor, descreva as condições assinaladas acima:**

**Altura: Peso: Pressão Arterial:**

**Você tem alergia a algum produto de higiene bucal, produto de higiene pessoal, ou algum dos seus ingredientes? ( ) Sim ( ) Não**

**Se houver, explique:** ________________________________________________________________________________________________________________________________________

Você faz o uso diário de algum medicamento?

| **Nome do medicamento** | **Dose** | **Frequência**  **diária** | **Motivo** |
| --- | --- | --- | --- |
|  |  |  |  |
|  |  |  |  |
|  |  |  |  |
|  |  |  |  |
|  |  |  |  |

**HISTÓRICO ODONTOLÓGICO**

**Nome do seu dentista:** ________________________________________________________

**Telefone do seu dentista:** _____________________________________________________

**Data da última visita ao dentista: ______________________________________________**

**Com que frequência você visita o seu dentista?** ___________________________________

**APENAS PARA MULHERES**

**Você está grávida?** ( ) Sim ( ) Não **Se estiver, de quantos meses? _____________**

**Atualmente, você está amamentando?** ( ) Sim ( ) Não

# APÊNDICE D. FORMULÁRIO DE EXAMES DE TECIDOS BUCAIS MOLES E DUROS

**Número do participante: Data: _/ __/**________

**Qual é o período experimental dessa avaliação?** ( ) Baseline ( ) 30 Dias ( ) 90 Dias

| Área | Normal | |  | |
| --- | --- | --- | --- | --- |
| 1. Palato mole | Sim ( ) | Não ( ) | |  |
| 2. Palato duro | Sim ( ) | Não ( ) | |  |
| 3. Mucosa gengival | Sim ( ) | Não ( ) | |  |
| 4. Mucosa bucal | Sim ( ) | Não ( ) | |  |
| 5. Pregas mucogengivais | Sim ( ) | Não ( ) | |  |
| 6. Língua | Sim ( ) | Não ( ) | |  |
| 7. Áreas sublingual e submandibular | Sim ( ) | Não ( ) | |  |
| 8. Glândulas salivares | Sim ( ) | Não ( ) | |  |
| 9. Áreas tonsilar e faríngea | Sim ( ) | Não ( ) | |  |

**Se alguma das respostas for “não”, detalhar:**

____________________________________________________________________________________________________________________________________________________________________________________________________________________________________________________________________________________________________________

#

**Data: __________ Assinatura do dentista examinador:____________________________**

# APÊNDICE E. FORMULÁRIO DE EXAME INICIAL

**Número do participante: ___________ Idade: ________Data: ____/____/_______**

1. O participante possui idade entre 18 anos e 70 anos? ( ) Sim ( ) Não
2. O participante tem disponibilidade para participar de todo o estudo? ( ) Sim ( ) Não
3. O participante apresenta boa saúde sistêmica? ( ) Sim ( ) Não
4. O participante está disposto a fornecer informações relacionadas ao seu histórico médico? ( ) Sim ( ) Não
5. O participante assinou o termo de consentimento livre e esclarecido? ( ) Sim ( ) Não

**Se, para as questões 1 a 5, houver alguma resposta “não”, o participante está inelegível para o estudo. Ele deverá ser dispensado, e a questão 14 deverá ser completada. Se o sujeito for elegível, preencha as questões 6-13.**

1. O participante está participando de outro ensaio clínico ou participou de algum ensaio clínico no último mês? ( ) Sim ( ) Não
2. A participante está grávida ou amamentando? ( ) Sim ( ) Não
3. A/O participante apresenta histórico de alergias a produtos de higiene bucal, produtos de higiene pessoal, ou a seus ingredientes? ( ) Sim ( ) Não
4. A/O participante, atualmente, está com irritação bucal ou utilizando anestésicos orais? ( ) Sim ( ) Não
5. A/O participante apresenta diabetes? ( ) Sim ( ) Não
6. A/O participante apresenta imunocomprometimento (HIV, AIDS, terapia medicamentosa imunossupressora)? ( ) Sim ( ) Não
7. A/O participante faz uso de prótese total? ( ) Sim ( ) Não
8. A/O participante apresenta síndrome do túnel do carpo ou artrite nas mãos? ( ) Sim ( ) Não

**Se, para as questões 6 a 13, houver alguma resposta “sim”, o participante está inelegível para o estudo. Ele deverá ser dispensado, e a questão 14 deverá ser completada**.

1. O sujeito está elegível a entrar no estudo? ( ) Sim ( ) Não

#

**Data: ________ Assinatura do dentista examinador: _______________________**

# APÊNDICE F. QUESTIONÁRIO DE REGISTRO DIÁRIO

| **Levantamento de Wisconsin sobre sintomas respiratórios de vias aéreas superiores (WURSS-21) - Diário de sintomas** |
| --- |
| Dia: Data: Horário: ID: |

Preencha **UM** círculo para cada item a seguir**:**

|  | Não me sinto doente  **0** | Muito levemente  **1** | **2** | Levemente  **3** | **4** | Moderadamente  **5** | **6** | Gravemente  **7** |
| --- | --- | --- | --- | --- | --- | --- | --- | --- |
| Quão doente você se sente **hoje**? |  |  |  |  |  |  |  |  |

Classifique a gravidade média de cada sintoma de resfriado em relação **às últimas 24 horas**:

|  | Não tem este sintoma  **0** | Muito leve  **1** | **2** | Leve  **3** | **4** | Moderado  **5** | **6** | Grave  **7** |
| --- | --- | --- | --- | --- | --- | --- | --- | --- |
| Coriza  (nariz escorrendo) |  |  |  |  |  |  |  |  |
| Nariz entupido |  |  |  |  |  |  |  |  |
| Espirro |  |  |  |  |  |  |  |  |
| Dor de garganta |  |  |  |  |  |  |  |  |
| Garganta arranhando  (coceira na garganta) |  |  |  |  |  |  |  |  |
| Tosse |  |  |  |  |  |  |  |  |
| Rouquidão |  |  |  |  |  |  |  |  |
| Pressão na cabeça |  |  |  |  |  |  |  |  |
| Pressão no peito |  |  |  |  |  |  |  |  |
| Cansaço |  |  |  |  |  |  |  |  |

**Caso você tenha apresentado ao menos UM dos sintomas acima, responda a seguir:**

**- Nas últimas 24 horas**, quanto o seu resfriado interferiu na sua capacidade de:

|  | De maneira alguma  **0** | Muito levemente  **1** | **2** | Levemente  **3** | **4** | Moderadamente  **5** | **6** | Gravemente  **7** |
| --- | --- | --- | --- | --- | --- | --- | --- | --- |
| Pensar com clareza |  |  |  |  |  |  |  |  |
| Dormir bem |  |  |  |  |  |  |  |  |
| Respirar com facilidade |  |  |  |  |  |  |  |  |
| Andar, subir escadas, exercitar |  |  |  |  |  |  |  |  |
| Realizar atividades diárias |  |  |  |  |  |  |  |  |
| Trabalhar fora de casa |  |  |  |  |  |  |  |  |
| Trabalhar dentro de casa |  |  |  |  |  |  |  |  |
| Interagir com as pessoas |  |  |  |  |  |  |  |  |
| Viver sua vida pessoal |  |  |  |  |  |  |  |  |

**- Comparado a ontem**, eu sinto que meu resfriado está:

| Muito melhor | Melhor | Um pouco melhor | Igual | Um pouco pior | Pior | Muito pior |
| --- | --- | --- | --- | --- | --- | --- |
|  |  |  |  |  |  |  |

# APÊNDICE G. RÓTULO DE INSTRUÇÕES PARA O PARTICIPANTE

Grupo Controle:

| **CRO-2022-04-FLU-REG-BZ-ZM**  **Product XXX**  *Escove os dentes duas vezes ao dia (pela manhã e à noite)*  *por dois minutos.*  *Não ingerir.*  *Uso restrito para pesquisa por participantes do estudo.*  *Somente para adultos. Manter fora do alcance de crianças.*  *Em caso de emergência, por favor telefonar para:*  ***Dr. Maísa Casarin – (Telefone omitido por motivos de privacidade)*** |
| --- |

Grupo teste:

| **CRO-2022-04-FLU-REG-BZ-ZM**  **Product XXX**  *Escove os dentes duas vezes ao dia (pela manhã e à noite) por dois minutos.*  *Após a escovação, gargarejar a boca com 20 ml de colutório, por 30 segundos, duas vezes ao dia*  *Não ingerir.*  *Uso restrito para pesquisa por participante do estudo.*  *Somente para adultos. Manter fora do alcance de crianças.*  *Em caso de emergência, por favor telefonar para:*  ***Dr. Maísa Casarin – (Telefone omitido por motivos de privacidade)*** |
| --- |

# APÊNDICE H. FORMULÁRIO DE VISITA

**Número do participante: Data: / /**

**Atualmente, o paciente faz uso de alguma medicação? (incluindo produtos de venda livre)**

**( ) Sim ( ) Não**

**Em caso afirmativo, liste todas as medicações:**

| **Medicação** | **Dose total diária** | **Data de início** | **Data do término (circule o “C” caso a medicação for de uso**  **contínuo** | **Indicação** |
| --- | --- | --- | --- | --- |
|  |  |  | **C** |  |
|  |  |  | **C** |  |
|  |  |  | **C** |  |
|  |  |  | **C** |  |

**Todas as medicações devem ser revisadas pelo supervisor do estudo.**

1. Houve alguma reação inesperada ou séria desde o exame anterior? ( ) Sim ( ) Não
2. Para a condição anteriormente citada, houve algum tratamento prescrito? ( ) Sim ( ) Não; Em caso afirmativo, descreva: ___________________________________________________________________________
3. Algum tratamento odontológico foi realizado desde o exame anterior? ( ) Sim ( ) Não;

Em caso afirmativo, descreva: ___________________________________________________________________________

1. Desde o último exame, houve a prescrição de alguma medicação? ( ) Sim ( ) Não;

Em caso afirmativo, descreva dose, duração e motivo: ___________________________________________________________________________

1. A voluntária está grávida ou amamentando? ( ) N/A ( ) Sim ( ) Não
2. Algumas das respostas das questões de 1 a 5 justificam a exclusão dos dados do participante da análise estatística? ( ) Sim ( ) Não;

Em caso afirmativo, explique: ___________________________________________________________________________

1. O participante continuará no estudo? ( ) Sim ( ) Não

**Se a resposta para a questão 7 for “Não”, complete a questão 8**.

1. O participante completou todo o estudo? ( ) Sim ( ) Não

Em caso negativo, explique: ______________________________________________________________________________________________________________________________________________________

**Data:** ______ **Assinatura do dentista examinador:** ______________________________
