# Supplementary material for: Efficacy of regular gargling with a cetylpyridinium chloride plus zinc containing mouthwash can reduce upper respiratory symptoms
Source: PLoS One. 2025 Feb 26;20(2):e0316807. doi: 10.1371/journal.pone.0316807 (PMC11864509; doi:10.1371/journal.pone.0316807)
Supplement: S2 Appendix — (DOCX) [file pone.0316807.s002.docx]

**FEDERAL UNIVERSITY OF PELOTAS**

**SCHOOL OF DENTISTRY**

**DEPARTMENT OF SEMIOLOGY AND CLINIC**

Research project

**EFFICACY OF CETYLPYRIDINIUM CHLORIDE AND ZINC MOUTHWASH IN REDUCING THE OCCURRENCE OF SYMPTOMS ASSOCIATED WITH THE FLU AND COLD SEASON – A RANDOMIZED CLINICAL TRIAL**

FRANCISCO WILKER MUSTAFA GOMES MUNIZ

Pelotas

May 2022

RESEARCH TEAM

FRANCISCO WILKER MUSTAFA GOMES MUNIZ – MsC and Ph.D. in Periodontology. Professor of Periodontology at the Federal University of Pelotas. Professor of the Postgraduate Program in Dentistry of Federal University of Pelotas.

CASSIANO KUCHENBECKER RÖSING – MsC and PhD in Periodontology. Professor of Periodontology at the Federal University of Rio Grande do Sul. Permanent Professor of the Postgraduate Program in Dentistry of Federal University of Rio Grande do Sul.

MAÍSA CASARIN – MsC and PhD in Periodontology. Professor of Periodontology at the Federal University of Pelotas. Professor of the Postgraduate Program in Dentistry of the Federal University of Pelotas.

NATÁLIA MARCUMINI POLA – MsC and PhD in Periodontology. Professor of Periodontology at the Federal University of Pelotas. Professor of the Postgraduate Program in Dentistry of the Federal University of Pelotas.

TACIANE MENEZES DA SILVEIRA – Master in Periodontology. PhD student in Dental Clinic/Periodontology of the Postgraduate Program in Dentistry of the Federal University of Pelotas.

GUILHERME AZÁRIO DE HOLANDA – PhD student in Dental Clinic/Periodontology of the Postgraduate Program in Dentistry of the Federal University of Pelotas.

FRANCISCO HECKTHEUER SILVA – MsC student in Dental Clinic/Periodontology of the Postgraduate Program in Dentistry of the Federal University of Pelotas.

LARISSA VIANA DE OLIVEIRA – Undergraduate dentistry student of the Federal University of Pelotas.

PEDRO PAULO DE ALMEIDA DANTAS – Undergraduate dentistry student of the Federal University of Pelotas.

**ABSTRACT**

The objective of this study will be to evaluate the efficacy of regular cleaning of the oropharynx on the incidence of symptoms associated with flu and colds. A total of 150 individuals will be randomized to one of the two experimental regimens and followed for a period of 90 days. The experimental regimes include: control, using toothpaste and a soft toothbrush; and test: using dentifrice, soft toothbrush, and mouthwash for gargling containing cetylpyridinium chloride (CPC) (0.075%) and zinc in an alcohol-free solution. All products are commercially available and will be provided by the research team. Participants will be instructed to brush their teeth twice a day for two minutes. Those allocated to the test regime will gargle with 20 ml of the mouthwash after each brushing. At the initial visit, participants will receive the products and instructions for use, as well as a daily log questionnaire. Participants will be asked to send their daily records once a week, using a messaging application. The proportion of days without any of the evaluated symptoms will be used as the main outcome. In addition, soft and hard tissue exams will be performed at the initial consultation, after 30 and 90 days of follow-up. Possible adverse events will be collected throughout the study. The groups will be compared using the chi-square test and the significance level will be set at 95%.

**Key-words:** Influenza, Oropharynx, Cetylpyridinium

**SUMMARY**

[1. INTRODUCTION 6](#_Toc170557057)

[2. AIM 8](#_Toc170557058)

[3. HYPOTHESIS 9](#_Toc170557059)

[4. METODOLOGY 10](#_Toc170557060)

[4.1. Experimental design and Place of Performance 10](#_Toc170557061)

[4.2. Study population 10](#_Toc170557062)

[4.2.1. Sample size 10](#_Toc170557063)

[4.2.2. Inclusion criteria 10](#_Toc170557064)

[4.2.3. Exclusion criteria 10](#_Toc170557065)

[4.3. Tests products 11](#_Toc170557066)

[4.4. Experimental procedure 11](#_Toc170557067)

[4.4.1. Selection of participants 11](#_Toc170557068)

[4.4.2. Soft and hard oral tissue exams 12](#_Toc170557069)

[4.4.3. Allocation of participants in each group 12](#_Toc170557070)

[4.4.4 Home use products and instructions for use to the participants 13](#_Toc170557071)

[4.4.5 Subsequent assessment 13](#_Toc170557072)

[4.5. Monitoring and discontinuity of participants 14](#_Toc170557073)

[4.6. Pregnancy 15](#_Toc170557074)

[4.7. Statistical analysis 15](#_Toc170557075)

[5 ETHICAL CONSIDERATIONS 15](#_Toc170557076)

[5.1. Approval by the Ethics Committee of Federal University of Pelotas 15](#_Toc170557077)

[5.2. Risks and benefits 16](#_Toc170557078)

[5.3. Study documentation 16](#_Toc170557079)

[6 HANDLING OF TEST PRODUCTS 17](#_Toc170557080)

[6.1. Test products 17](#_Toc170557081)

[6.2. Storage, handling and accountability of test products 17](#_Toc170557082)

[6.3. Administration of the test products 18](#_Toc170557083)

[7. CONCOMITANT THERAPY 18](#_Toc170557084)

[8. ADVERSE EVENTS 19](#_Toc170557085)

[8.1. Definitions 19](#_Toc170557086)

[8.2. Reporting and documentation of adverse events 20](#_Toc170557087)

[8.3. General procedures for all adverse events 20](#_Toc170557088)

[9. NEW FINDINGS 21](#_Toc170557089)

[REFERENCES 22](#_Toc170557090)

[SCHEDULE 24](#_Toc170557091)

[BUDGET 26](#_Toc170557092)

[APPENDIX A – FREE AND INFORMED CONSENT TERMS 28](#_Toc170557093)

[APPENDIX B – ADVERSE REACTIONS FORM 34](#_Toc170557094)

[APPENDIX C – HEALTH QUESTIONNAIRE 37](#_Toc170557095)

[APPENDIX D – SOFT AND HARD ORAL TISSUE EXAMINATION FORM 40](#_Toc170557096)

[APPENDIX E – INITIAL EXAM FORM 41](#_Toc170557097)

[APPENDIX F – DAILY LOG QUESTIONNAIRE 42](#_Toc170557098)

[APPENDIX G – INSTRUCTIONS LABEL FOR PARTICIPANT 45](#_Toc170557099)

[APPENDIX H – VISIT FORM 46](#_Toc170557100)

# **INTRODUCTION**

Respiratory infections of viral origin are responsible for high degrees of morbidity and mortality around the world. Among viral respiratory infections, those caused by the Influenza virus are one of the most common. Furthermore, its impact on health systems is of great importance (FRASER, TOMBE-MDEWA, KOHLI-LYNCH, *et al*., 2022).

Since the emergence of the COVID-19 pandemic, interest in viral respiratory infections has grown, and a series of studies have been performed to understand the role of different strategies that could mitigate the effects of viral respiratory infections. For both influenza and COVID-19 infections, vaccination has proven to be a preventive and damage limitation strategy with satisfactory degrees of efficiency, from individual and, mainly, collective aspects.

However, in addition to vaccination, different strategies have been proposed. Specifically in the case of the oral cavity, studies have been performed with different agents previously used with the aim of supporting oral hygiene and have been revisited in relation to their antiviral capacity (FERNANDEZ, GUEDES, LANGA, et al., 2022), with a view to determining possible/potential effects on viral respiratory infections, considering that the oropharynx is one of the affected spaces and a potential entry point for the infection. However, much of this literature comes from *in vitro* studies, and there is a gap in the literature regarding the clinical antiviral efficacy of these oral hygiene products.

Similarly to hand washing/hygiene, which has been considered an impactful measure in preventing the spread of viral infections, it is hypothesized that oral and oropharyngeal hygiene measures may also have such beneficial effects. Within this context, clinical trials that verify the clinical efficacy of these agents are necessary.

In this sense, mouthwash solutions have been studied in relation to their antiviral potential. When chlorhexidine-based solutions are evaluated, in a systematic review of the literature, it is observed that they may have an interesting virucidal effect on influenza viruses and even SARS-COV 2 (FERNANDEZ, GUEDES, LANGA, et al., 2022).

Other studies have been carried out and sought to evaluate the potential of solutions used locally in the mouth/oropharynx region in the context of viral infections. In an *in vitro* study, using two commercially available toothpastes and two mouthwash solutions, it was observed that rinsing with hydrogen peroxide, or with cetylpyridinium chloride, or using toothpastes with stannous fluoride had important antiviral effects (RAMJI, CIRCELLO, WINSTON, et al., 2022).

Cetylpyridinium chloride (CPC) is a quaternary ammonium compound, with high safety and widespread use as an adjunct to oral hygiene procedures. It has good adherence by patients, and its antibacterial effect on the oral microbiota is undisputed (LANGA, MUNIZ, COSTA, et al., 2021). Furthermore, recently, the literature has demonstrated that the addition of zinc (Zn) to mouthwashes containing CPC has demonstrated superior clinical effects when compared to mouthwashes containing only CPC (RÖSING, CAVAGNI, GAIO et al., 2017) or essential oils (LANGA, CAVAGNI, MUNIZ et al., 2021). However, its antiviral potential is still little tested, but it has potential and has been recommended as a pre-operative mouthwash for dental procedures (RETAMAL-VALDES, SOARES, STEWART, et al., 2017).

Based on these potentials and the need for clinical studies, in addition to the possible effect of the topical use of oral antiseptics to reduce viral infections, this study is justified, which aims to evaluate the effect of using a CCP+Zn solution in the form gargling when flu and cold symptoms occur. The hypothesis under study is that the local presence of CCP+Zn can reduce the occurrence of flu-like symptoms when compared to the absence of these active ingredients.

# **AIM**

The aim of this study will be to evaluate the efficacy of regular cleaning of the oropharynx, on the incidence and duration of symptoms associated with flu and colds, with the use of CCP+Zn gargling compared to the absence of gargling.

# **HYPOTHESIS**

The operational hypothesis is that there will be a significant reduction in symptoms associated with flu and colds after using the CCP+Zn-based product, compared to the experimental control regime.

# **METODOLOGY**

# **4.1. Experimental design and place of performance**

This is a phase III, randomized, controlled, parallel, single-center clinical trial to evaluate the efficacy of regular oropharyngeal cleaning on the incidence and duration of symptoms associated with cold and flu. The study will be carried out in the dental clinics of the Faculty of Dentistry of the Federal University of Pelotas (UFPel).

## **4.2. Study population**

One hundred and fifty (150) individuals, aged 18-70 years, will be included in this study.

### Sample size

The sample size of 150 individuals (75 per group) was based on internal data from the sponsor of this study. It was determined that 60 individuals per group are needed for 80% power for the occurrence of flu-like symptoms. Considering the nature of the study and the demand for daily questionnaire responses, an attrition rate of 25% is assumed over the course of the study.

- - 1. Inclusion criteria

Individuals must meet all the following criteria:

1. Sign this informed consent form (ICF) (Appendix A);

2. Men or women, aged between 18 and 70;

3. Good systemic general health as determined by study investigators;

4. Availability of 90 days to participate in the study;

- - 1. Exclusion criteria

During the study, the following exclusion criteria will be considered:

1. Be participating in any other clinical study;

2. Being pregnant or breastfeeding;

3. Present a history of allergies to oral hygiene products, personal hygiene products, or their ingredients;

4. Have mouth irritation or use oral anesthetic sprays;

5. Have diabetes;

6. Be undergoing extensive dental treatment or oral surgery during the study;

7. Present immunocompromised (HIV, AIDS, immunosuppressive drug therapy);

8. Use complete dentures;

9. Have carpal tunnel syndrome or arthritis in the hands.

10. Participant who substantially fails to follow the required protocols;

11. Participant who fails to attend scheduled appointments;

12. Participant who is treated, during the study period, with medications that may interfere with the parameters being analyzed in the study;

13. Participant who is treated by a medical or dental service, and this may interfere with the parameters being analyzed in the study;

14. Participant who develops serious adverse reactions. The investigator will immediately record the information on the Adverse Reactions Form (Appendix B);

15. The participant who chooses to end their participation in the study;

16. Participant reports being pregnant during the study.

- 1. **Tests products**

Participants will be randomized into one of the following experimental regimens and will receive products as follows:

Group control:

● Colgate Maximum Cavity Protection® Toothpaste.

● Commercially available soft toothbrush.

Test Group:

● Mouthwash containing 0.075% CCP and 0.28% zinc lactate, in an alcohol-free base, commercially available in Brazil.

● Colgate Maximum Cavity Protection® Toothpaste.

● Commercially available soft toothbrush.

## **4.4. Experimental procedure**

### 4.4.1. Selection of participants

Research participants who sign the informed consent form and answer to the health questionnaire (Appendices A and C) will receive an intraoral clinical examination to identify whether they meet the inclusion and exclusion criteria. All individuals will receive an assessment of their soft and hard oral tissues, findings will be recorded on the Hard and Soft Buccal Tissue Form (Appendix D). The initial questionnaire will be recorded on the Initial Examination Form (Appendix E). The first 150 individuals who meet the inclusion criteria, sign the consent form and the health questionnaire will be included in the study. After initial assessments, all individuals will receive a questionnaire to record daily symptoms associated with the flu and cold (Appendix F). This form will be delivered in physical format, with the questionnaire for the 90 days of the study, to facilitate completion by the participant. Once a week, participants must send photographs of their daily records. This will be sent via a messaging application. Participants will be reimbursed amounts compatible with internet packages. The research team undertakes to download the records and store them on devices on external physical drives, not connected to the internet. Messages will be deleted immediately after downloads. Furthermore, confidentiality of participants' data will be ensured throughout data storage and other procedures of this study.

The selection of participants will be of convenience based on a verbal invitation at the different clinics of the Dentistry Faculty of Federal University of Pelotas. In addition, recruitment may include posts on social media, which explain the main objectives of the present study. Those who express interest in participating in the study will receive the consent form in two copies and, after reading and signing it, will be evaluated based on the following exams:

4.4.2. Soft and hard oral tissue exams

All individuals will receive an examination of the soft and hard oral tissues. This exam will be performed by a single trained and calibrated examiner. With the aid of an odontoscope and artificial lighting from the dental chair, the examination will include an evaluation of the hard and soft palate, gingival mucosa, buccal mucosa, mucogingival areas, tongue, sublingual and submandibular areas, salivary glands and tonsillar, and pharyngeal areas. Findings will be recorded on the Soft and Hard Tissue Examination Form (Appendix D). If necessary, individuals will be immediately referred to the Stomatology Service of the Faculty of Dentistry at Federal University of Pelotas.

### 4.4.3. Allocation of participants in each group

Participants included will be randomly allocated to one of the two study groups, via a website (randomization.org). The examining dental surgeon and his assistant will not receive information, throughout the study, regarding the allocation group of each participant. The products will be covered by a wrapper to maintain allocation concealment. The products will be distributed by a researcher not involved in the clinical evaluations, in a separate area from the examination and interview site and placed in a sealed bag to avoid the perception of any differences in the packaging and appearance of the products used between the two experimental groups. Label information will consist of a study group code, instructions for home use, and safety information including emergency contacts (Appendix G).

Participants will be numbered, as a form of identification, chronologically from 001 to 150, as they are included in the study. Participants living in the same house will be assigned to the same experimental group, that is, they will receive the same products.

At the initial visit, as a form of guidance, participants will be instructed to practice oral hygiene according to the experimental regimen designated by a member of the research team. In the case of the control regime, participants will be asked to brush their teeth for 2 minutes with toothpaste twice a day. In the test regime, in addition to brushing for two minutes with toothpaste, participants must gargle with the substance offered for 30 seconds, twice a day.

### 4.4.4 Home use products and instructions for use to the participants

Following the dental evaluation, all subjects will receive products according to the designated regimen for home use:

Control group: will receive a toothbrush with soft bristles for adults and a toothpaste. Instructions for use: brush your teeth for two minutes, twice a day (morning and evening) with the toothpaste provided.

Test group: will receive a toothbrush with soft bristles for adults, mouthwash and toothpaste. Instruction for use: brush your teeth for 2 minutes, twice a day (morning and evening) with the toothpaste provided. After brushing, gargle with mouthwash (20 ml) for 30 seconds, twice a day (morning and evening), and then spit it out.

Participants will be instructed to perform the procedures described above and only use the product assigned to their group during the study period. Products will be provided additionally on subsequent visits. There will be no restrictions on eating habits during the study, nor cleaning between teeth. Upon completion of the study, participants will be instructed to return all used products.

### 4.4.5 Subsequent assessment

Subsequent evaluations will be performed after 30 and 90 days of use of the designated products. Participants will attend the research location to be evaluated and receive additional products, according to the group to which they were allocated. During these visits, an Oral Assessment of Soft and Hard Tissues will be carried out (Appendix D). A Visit Form (Appendix H) will be completed for all study participants.

After the 90 days of the experiment, all participants will be instructed to no longer use the products provided, returning to their normal oral hygiene habits. All unused products will be handed over to an assistant not involved in the clinical examination and will be discarded following Federal University of Pelotas waste disposal guidelines.

- 1. **Monitoring and discontinuity of participants**

All randomized research participants who receive the products will be monitored according to the following protocol:

Participants will be considered to have completed the study if they are re-evaluated at the end of 90 days. They will only be considered lost to follow-up if no contact was established during the completion of the study, and there is, therefore, insufficient information to determine the status of the research participant. Efforts will be made to determine the reasons why the participant did not return for the required visit(s) or the reasons for the participant's withdrawal from the study. Participants will exit the study if any of the following occur:

1. Participant who substantially fails to follow the required protocols;

2. Participant who fails to attend scheduled appointments;

3. Participant who is treated, during the study period, with medications that may interfere with the study parameters being analyzed;

4. Participant who is treated by a medical or dental service, and this may interfere with the parameters being analyzed in the study;

5. Participant who develops serious adverse reactions. The investigator will immediately record the information on the Adverse Reactions Form (Appendix B);

6. The participant who chooses to end their participation in the study;

7. Participant reports being pregnant during the study.

The researcher will be responsible for promptly notifying the Federal University of Pelotas Research Ethics Committee about all participants who leave the study prematurely and experience serious adverse effects.

## **Pregnancy**

There is no intention to include pregnant women in the present study. If any woman becomes pregnant during this clinical study, her participation will be terminated and there will be a notification of the event by the responsible researcher.

## **4.7. Statistical analysis**

Chi-square test will be performed for gender data, and an independent t-test for the age variable, to test the hypothesis that the groups present balanced distribution of variables. The proportion of symptom-free days (i.e., none of the 10 symptoms were present) will be used for the primary outcome variable. Chi-square test will be used to compare the proportion of symptom-free days for the experimental groups. Analysis of variance can be used to compare groups. Significance level will be set at 95%.

# **ETHICAL CONSIDERATIONS**

## **5.1. Approval by the Ethics Committee of Federal University of Pelotas**

This research project will be submitted for evaluation by the Federal University of Pelotas Research Ethics Committee (REC), via Plataforma Brasil, and will only begin after approval. The study will be conducted in accordance with the standards of “Good Clinical Practice”. All research participants will sign a consent form (Appendix A).

It should be noted that the product to be tested already has a similar formulation in different products sold on the Brazilian market. In this sense, it is important to point out that none of the products to be used in the study are new products to be commercialized.

## **5.2. Risks and benefits**

In general, it is not expected that there will be any side effects from the use of any of the products under test, considering that they are products in routine use and commercially available. However, there is the possibility of soft tissue irritation or temporary tooth sensitivity with the use of these products, as with any oral hygiene product that is regularly used. When these conditions occur, it is recognized that they disappear with the suspension of use of the product. The oral tissue examination, which will be performed, is used as part of routine oral health care. There is also a risk of discomfort when answering the questionnaire questions. Participants will need to spend time traveling and participating throughout the study period, in addition to the need to purchase a mobile data package for the weekly questionnaires sent out. Because of this, patients will be compensated with the amount of R$50.00 (fifty Brazilian Reais), for each visit carried out, in order to offset the expenses inherent to their travel to the Faculty of Dentistry at Federal University of Pelotas. In addition, the purchase of the mobile data package will also be reimbursed with the amount of R$100.00 (one hundred Brazilian Reais) during the participants' last return.

No direct benefits are expected from participating in this study. The results of the study may help to find alternative methods to reduce the occurrence of flu and cold symptoms. Additionally, help ensure that the product is safe to market to the public. The research team will be responsible for dealing with adverse events arising from participation in the study.

## **5.3**. **Study documentation**

All data relevant to the assessments outlined in this protocol must be recorded on the forms provided. A code will be generated for each participant at the beginning of the study and this will be used in all other forms throughout the experimental period, with a view to guaranteeing anonymity. Upon completing the form, the investigator or assistant must sign the document to accept responsibilities for the purpose of recording data on each page of the form.

The following participant registration forms will be completed by the study researcher according to the following sequence:

**Initial visit**

● Consent form (Appendix A)

● Health Questionnaire (Appendix C)

● Initial Examination Form (Appendix E)

**CLINICAL EVALUATIONS**

● Examination of Soft and Hard Oral Tissues (Appendix D)

**Visit 2: day 30**

● Visit Form (Appendix H)

**CLINICAL EVALUATIONS**

● Examination of Soft and Hard Oral Tissues (Appendix D)

**Visit 3: Day 90**

● Visit Form (Appendix H)

● Delivery of the set of physical questionnaires answered during the study (Appendix F)

**CLINICAL EVALUATIONS**

● Examination of Soft and Hard Oral Tissues (Appendix D)

# **HANDLING THE TEST PRODUCTS**

## **6.1. Test products**

All test products will be supplied by Colgate-Palmolive and will be handled in accordance with the biosafety standards in force at Dentistry Faculty of Federal University of Pelotas.

## **Storage, handling and accountability of test products**

The test products will be stored in a safe area, with limited access and at room temperature. The investigator will be directly responsible for the accommodation of all products used or not used in the study. The examiner and assistant will remain blind to the treatment used for each participant. Records will be maintained to document the recipe and disposition of all products used in the study, provided by the investigator to the researcher.

## **Administration of the test products**

All participants will receive their assigned test products at initial consultation/inclusion. Participants will receive written instructions on how to use the products.

● Test product dosage: All treatments will be distributed by the study coordinator at the study location.

● Time and place of product dispensing: it will be carried out on the day/time of each participant's inclusion in the dental clinic.

● Storage of agents between appointments: All products will be kept in a closed clinical area.

# **CONCOMITANT THERAPY**

If any participant takes concomitant drug therapy, as a necessity for the treatment of a medical condition, then the participant's maintenance during the experimental period of the study will be evaluated and defined at the discretion of the investigator. However, it is the investigator's responsibility to disqualify the entry of any individual who, during initial assessments, is using or consuming products that may hinder the interpretation of study results. All medications used by participants, on the day of inclusion, or at any time during the study period, will be recorded on the Visit Form (Appendix H). Participants may be given medications to treat adverse effects as necessary by the investigator or the participant's physician.

# **ADVERSE EVENTS**

## **Definitions**

Adverse events (AEs) and serious adverse events (SAEs) are defined by the Good Clinical Practice Guidelines as follows:

**Adverse events:** Any unconventional medical occurrence in a patient or in clinical research participants who are receiving administration of a pharmacological agent and which does not necessarily need to have a causal relationship with the treatment. An adverse event can, therefore, be any unfavorable and unintended sign (including abnormal laboratory findings), symptoms or temporary illnesses associated with the use of medicinal products, whether or not related to the medicinal product.

AEs include any sign of deterioration in a participant's medical status after being enrolled in a study. AE can involve any organ or system that can be represented by the new occurrence or deterioration of the disease, a syndrome, a symptom, a physical sign, as well as by findings and results of instrumental examinations and laboratory tests. Any inconvenient change since the subject's inclusion, occurring after the first administration of the study test product, is considered an adverse effect. All such occurrences must be recorded and reported appropriately, whether they are related to the study medication or not.

## **Reporting and documentation of adverse events**

All adverse event forms are provided in Appendix B.

## **General procedures for all adverse events**

All complaints, symptoms or signs that meet the definitions of an adverse effect must be recorded on the Adverse Reactions Form (Appendix B), including the following:

• Description of the adverse event

• Start date of the reaction

• Resolution date

• Outcome

• Severity

• Seriousness

• Relationship to study drug (causality)

• Actions taken

Adverse events will be assessed by the research team in terms of severity, relationship with the product under study, and possible etiologies. The case report form proposes that all recorded adverse event outcomes be reported within two weeks after the end of the study. The investigator is responsible for tracking all adverse effects until resolution or until there are no longer any clinical concerns and providing this data to the funder. The investigator is also responsible for reporting all adverse events to Ethical Committee Research – Federal University of Pelotas, in accordance with the rules and procedures established by resolution 466/12.

# **NEW FINDINGS**

Participants will be informed of any new significant findings related to study products or procedures as they become known during the course of this clinical study.

Such information may affect the subject's decision to continue participation in the study.

# **REFERENCES**

FRASER, H.; TOMBE-MDEWA, W.; KOHLI-LYNCH, C.; HOFMAN, K.*et al.* Costs of seasonal influenza vaccination in South Africa. **Influenza Other Respir Viruses**, Mar 30 2022.

FERNANDEZ, M. D. S.; GUEDES, M. I. F.; LANGA, G. P. J.; RÖSING, C. K.*et al.* Virucidal efficacy of chlorhexidine: a systematic review. **Odontology**, 110, n. 2, p. 376-392, Apr 2022.

RAMJI, N.; CIRCELLO, B.; WINSTON, J. L.; BIESBROCK, A. R. Virucidal Activity of Over-the-Counter Oral Care Products Against SARS-CoV-2. **Oral Health Prev Dent**, 20, n. 1, p. 185-192, Apr 27 2022.

LANGA, G.P.J.; CAVAGNI, J.; MUNIZ, F.W.M.G.; OBALLE, H.J.R., *et al.* Antiplaque and antigingivitis efficacy of cetylpyridinium chloride with zinc lactate compared with essential oil mouthrinses: Randomized clinical trial. **J Am Dent Assoc**, 152, p. 105-114, Feb 2021.

LANGA, G. P. J.; MUNIZ, F.; COSTA, R.; DA SILVEIRA, T. M.*et al.* The effect of cetylpyridinium chloride mouthrinse as adjunct to toothbrushing compared to placebo on interproximal plaque and gingival inflammation - a systematic review with meta-analyses. **Clin Oral Investig**, 25, n. 2, p. 745-757, Feb 2021.

RETAMAL-VALDES, B.; SOARES, G. M.; STEWART, B.; FIGUEIREDO, L. C.*et al.* Effectiveness of a pre-procedural mouthwash in reducing bacteria in dental aerosols: randomized clinical trial. **Braz Oral Res**, 31, p. e21, Mar 30 2017.

RÖSING, C.K.; CAVAGNI, J.; GAIO, E.J.; MUNIZ, F.W.M.G.; *et al.* Efficacy of two mouthwashes with cetylpyridinium chloride: a controlled randomized clinical trial. **Braz Oral Res**, 31, p. e47, Jul 2017.

# **SCHEDULE**

The current research project is expected to begin in July 2022. However, this project will only begin after approval by the Research Ethics Committee of the Faculty of Dentistry at Federal University of Pelotas.

| **Activity/Month**  **(after approval of Ethical Committee)** | **Month 1** | **Month 2** | **Month 3** | **Month 4** | **Month 5** | **Month 6** | **Month 7** | **Months**  **8 - 12** |
| --- | --- | --- | --- | --- | --- | --- | --- | --- |
| Literature research | **X** | **X** | **X** | **X** | **X** | **X** | **X** |  |
| Examiner training | **X** |  |  |  |  |  |  |  |
| Selection of participants and data collect | **X** | **X** | **X** | **X** | **X** |  |  |  |

| Data tabulation and analysis |  |  |  |  | **X** | **X** | **X** |  |
| --- | --- | --- | --- | --- | --- | --- | --- | --- |
| Writing of articles, preparation of the research report and submission for publication |  |  |  |  |  |  | **X** | **X** |

# **BUDGET**

This study has full funding from the Latin American Association for the Promotion of Oral Health and Dental Research – LAOHA.

| **Product** | **Unit price (R$)** | **Amount** | **Total (R$)** |
| --- | --- | --- | --- |
| Transportation costs | 50.00 | 450 | 22,500.00 |
| Cellphone internet plan reimbursement | 100.00 | 150 | 15,000.00 |
| Book – daily questionnaire | 30.00 | 150 | 4,500.00 |
| Copy of the questionnaires | 0.10 | 3,375 units | 337.50 |
| Copy of the informed consent forms | 0.10 | 400 units | 40.00 |
| Paper prints | 0.10 | 1000 units | 100.00 |
| Dentifrice (control regime) | 10.00 | 40.5kg | 4,500.00 |
| Dentifrice (test regime) | 10.00 | 40.5kg | 4,500.00 |
| Soft multi-bristle toothbrushes | 5.00 | 400 units | 2,000.00 |
| Mouthwash (CPC+Zn) | 15.00 | 450 units | 6,750.00 |
| Odontoscope, clinical forceps, millimiter periodontal probe | 70.00 | 30 units | 2,100.00 |
| Individual protective equipment (gloves, mask and hat) | - | - | 500.00 |
| Office supplies (pens, pencils, eraser), printer ink (existing printer) | - | - | 380.00 |
|  | | **Total** | **63,207.50** |

**APPENDIX A – FREE AND INFORMED CONSENT TERMS**

Dear Collaborator, you are being invited to participate in the following study:

**Research title: Efficacy of cetylpyridinium chloride and zinc mouthwash in reducing the occurrence of symptoms associated with the flu and cold season – a randomized clinical trial**

**Responsible researcher: Prof. Dr. Francisco Wilker Mustafa Gomes Muniz**

**Institution to which the responsible researcher belongs: Federal University of Pelotas**

**Location of study/data collection: School of Dentistry**

Dear participant,

You are being invited to participate in a clinical study lasting 90 (ninety) days. This informed consent aims to give you the information you will need to decide whether to participate in the study. Please read this consent form carefully. You can ask about the purpose of this research, what you will be asked to do, the possible risks and benefits, your rights as a participant, and any other questions about the research or consent that are not clear. After we answer all your questions, you can decide whether or not you want to participate in the study.

1. **RESEARCH AIM:** The aim of this study is to evaluate the efficacy of commercially available oral hygiene products on the occurrence of symptoms associated with colds and flu. With the results of this study, it is possible to find methods that can help reduce the occurrence of symptoms associated with flu and colds. This research project was evaluated by the Federal University of Pelotas Research Ethics Committee, a collegiate body, of a consultative, deliberative and educational nature, whose purpose is to evaluate – issue an opinion and monitor research projects involving human beings, in their ethical and methodological aspects, carried out at the scope of the institution.

2. **PARTICIPATION IN THE RESEARCH:** Your participation in the research will take place for a total of 3 (three) visits to the study site, over the 90 (ninety) days duration. If you meet the criteria for participation at the initial visit (visit 1), you will receive your assigned oral care products.

At the start of the study (visit 1), you will attend the clinic where the study researcher will perform an initial screening to determine if you are eligible to participate in the study. You will be randomly assigned to one of two study regimens: use of a soft toothbrush + toothpaste OR use of a soft toothbrush + toothpaste + mouthwash to gargle. Products will be given to use at home. Your information (age, date of birth, sex, initials of your name, etc.) will be collected. You will complete a health questionnaire about your medical history, dental history, oral health conditions, and current medications. An oral tissue examination will be performed to check for abnormalities. You will receive a questionnaire to complete at home daily, where you will record your symptoms associated with the flu and cold. Once a week (every Monday), you must send photos via Whatsapp of the previous week's questionnaires.

You will be instructed about the treatment requirements of this study. You will be asked to brush your teeth with the toothbrush and paste provided twice a day, morning and evening, for two (2) minutes. If you also receive mouthwash, you will be asked, after brushing your teeth, to gargle with 20 mL (twenty) of this product for 30 (thirty) seconds. At the end you must spit out the product. This product, mouthwash, will be used twice a day, always after brushing. The products must be used throughout the study period, 90 (ninety) days, and no other products can be used. The active ingredient to be tested in this research is already used in other products.

We would like to clarify that your participation is completely voluntary, and you are free to refuse to participate, or even withdraw at any time, and demand the withdrawal of your participation in the research without this causing any burden or harm to you.

Please remember: during this research study, you may not use dental products other than those provided (including toothpastes and toothbrushes, mouthwash, breath chews, etc.), or have your teeth cleaned or whitened in a dental office. You must only use the trial products given to you throughout this study. There are no restrictions on diet during the study. Routine dental treatments should not be performed; however, emergencies can be performed. Please inform the examining dentist if you have received emergency dental treatment, taken any new medications, received dental treatment, become pregnant or breast-fed.

3. **RESEARCH LOCATION:** You will need to attend the Federal University of Pelotas, Faculty of Dentistry (Gonçalves Chaves Street, 457 – Pelotas, Rio Grande do Sul) to carry out the three dental consultations, as previously reported, which may take approximately 20 minutes.

4. **RISKS AND DISCOMFORTS:** In general, no side effects are expected to occur from the use of any of the test products. However, there is the possibility of soft tissue irritation or temporary hypersensitivity with the use of these products, as with any oral care product you regularly use. These reactions are not harmful and disappear when treatment is stopped. When these conditions occur, suspend use of the products and contact the researcher for guidance. The oral tissue examination that will be performed is used as part of routine oral health care. There is also a risk of discomfort when answering the questionnaire questions. You will need to spend time traveling and participating throughout the study period.

Remember: All products you receive will be for your use only. Keep all products out of the reach of children and people unable to read or understand labels, as well as pets.

If you have any problems, you should contact Dr. Francisco Wilker Mustafa Gomes Muniz, on the phone provided in the clinical setting. If you are unable to communicate, please see your doctor. Any expense resulting from a problem arising from the use of the test products will be covered by the investigators.

If you suffer any harm as a result of participating in the study, you have the right to full, immediate and free assistance (responsibility of the researchers) and you also have the right to seek compensation if you feel that there has been any type of abuse by the researchers.

1. **BENEFITS**: Participation in this study may not directly benefit you. The results of

the study, however, can help to find alternative methods that can help reduce the occurrence of cold and flu symptoms in the population. Study results can also help ensure that the product is safe to market to the general public.

6. **CONFIDENTIALITY**: All information that you provide to us or that is obtained through interviews and clinical dental examination will only be used for this research. Your answers, personal data and dental and flu-symptom assessments will be kept confidential and your name will not appear anywhere on the questionnaires used. When the research results are released, this will be in coded form, to protect your name and maintain your confidentiality.

Your participation data in this study is confidential. Your medical and dental records will be maintained in accordance with Resolution 466/12, which guarantees the privacy and confidentiality of your data. Furthermore, signing this term does not exclude the possibility of seeking compensation for any damages arising from participation in this research.

The records of this study will be stored for a period of 5 (five) years, in a properly restricted location, to which only the main researcher will have access on a password-protected computer.

The results of this study may be published in a scientific journal, but its name will not be revealed.

Your signature below means that you understand and agree to the information above, and affirms that you volunteer to participate. Furthermore, you affirm that you responded truthfully, that you were given the opportunity to ask questions about the study, and that you received a copy of this consent form.

7. **EXPENSES/REPAIRS:** The project costs are the responsibility of the researcher. You will be reimbursed fifty Brazilian reais (R$50.00) for each of the three study visits, to cover transportation and food expenses. Furthermore, on your third visit, you will be reimbursed one hundred Brazilian reais (R$100.00) to cover your internet plan expenses throughout the entire study period.

8. **MATERIALS**: After 30 (thirty) and 90 (ninety) days of using the products, you will attend the dental clinic. At these two visits, the research team will update your medical/dental history to check for any changes in your health, or new medications since your previous study visit. You will be asked questions to find out whether you used the products correctly and any problems regarding the use of the products. The oral soft tissues will also be examined at these two visits. Completion of the daily questionnaire you received will be checked.

At the end of the 90-day study period, you will stop using the designated products and return to your oral hygiene routine. All remaining products must be returned on the final visit (day 90). These products cannot be shared with anyone in your family.

If you have further questions or require further clarification, you can contact us at the addresses below or contact the Research Ethics Committee of the Faculty of Dentistry at Federal University of Pelotas, whose address appears in this document.

The Ethics Committee, in accordance with Resolution 466/2012-CNS-MS, is an interdisciplinary and independent collegial body, of a consultative, deliberative and educational nature, created to defend the interests of research participants in their integrity and dignity and to contribute in the development of research within ethical standards. To ensure ethical standards in research, the previous topics provide minimum requirements to maintain your integrity and dignity in research.

As legal security, this term must be completed in two copies of equal content, one of which must be duly completed and signed and delivered to you. In addition to the signature in the specific fields by the researcher and you, we request that all pages of this document be initialed. This must be done by both (the researcher and you, as the research participant) in order to guarantee access to the complete document.

You may contact the responsible researcher or the Research Ethics Committee, using the information, addresses and telephone numbers contained below.

I, __________________________________________________________________ declare that I have been duly informed and agree to participate VOLUNTARILY in the research coordinated by **Prof. Dr. Francisco Wilker Mustafa Gomes Muniz**

_____________________________________________ Date: _______________

Signature or typescript of the employee

I, Francisco Wilker Mustafa Gomes Muniz, declare that I provided all the information regarding the aforementioned research project.

________________________________________ Date: _______________

Signature of the researcher

• Any doubts regarding the research can be clarified with the researcher, according to the details and address below:

Name: Francisco Wilker Mustafa Gomes Muniz

Address: Gonçalves Chaves Street, 457

Email: [wilkermustafa@gmail.com](mailto:wilkermustafa@gmail.com)

• Any doubts regarding the ethical aspects of the research can be clarified

with the Research Ethics Committee of the Faculty of Dentistry at Federal University of Pelotas, at the address below:

Research Ethics Committee of the Faculty of Dentistry of the Federal University of Pelotas

Telephone: +555332602801

Email: cepodonto@ufpel.edu.br

Address: Gonçalves Chaves Street, 457, Pelotas, Rio Grande do Sul, Brazil.

OPENING HOURS:

Monday to Friday, from 8am to 11:30am and from 2pm to 5:30pm

**APPENDIX B – ADVERSE REACTIONS FORM**

**Information’s about the participant:**

| Number of  Participant | Initials of  participante | Gender | Age | Weight | Ethnic group |
| --- | --- | --- | --- | --- | --- |
|  |  |  |  |  |  |

If the participant is a woman, are they pregnant? ( ) Yes No

If yes, how many months? _________

Date the adverse reaction was noticed: ______/______/________

Adverse reaction reporting date: ______/______/_________

Phase of the study where the first event occurred: ___________________________________

**Information’s about the product used:**

| Nome of the product (identification number): | |
| --- | --- |
| Data of first usage: | Date of interruption usage: |
| Dose: | Daily frequency: |
| Experimental group: | |

**Information’s about adverse reactions:**

| **Start date** | **Duration or end date** | **Severity (mild, moderate, severe)** | **Relationship with the product (possible related; unrelated; unknown)** |
| --- | --- | --- | --- |
|  |  |  |  |

**Describe the adverse reaction in details: ______________________________________________________________________________________________________________________________________________________________________________________________________________________________________________________________**

**Regarding the adverse reaction, answer:**

( ) Resolved – Date:____/____/______

( ) Resolution process

( ) Unknown/Lost to follow-up

( ) Not solved

( ) Resolved with sequel

( ) Other: ________________________

**Action performed with the test product:**

( ) Continued use

( ) Stopped use

( ) Stopped use temporarily

( ) Reduced use, specify:_____________________________________________

( ) Unknown

( ) Other

**Did the adverse reaction decrease after stopping or reducing the dose of the product?**

( ) Yes No

**Did the adverse reaction reappear after reintroducing use of the product?**

( ) Yes No

**Participant protocol status:**

( ) Continuous protocol

( ) Discontinuous protocol

**Treatment for adverse reaction/serious adverse reaction:** ____________________________________________________________________________________________________________________________________________

**Relevant medical history data:**

( ) Yes, list them ( ) None ( ) Not provided ( ) Unknown

- Medical history, with start date, if known: ______________________________________________________________________

**Relevant concomitant medications:**

( ) Yes, list them ( ) None ( ) Not provided ( ) Unknown

- Medication name, dose, frequency, medication start and stop dates or

duration of therapy if known is: _____________________________________________________________________

**Relevant laboratory data:**

( ) Yes, list them ( ) None ( ) Not provided ( ) Unknown

- Laboratory test, results and dates if known: ______________________________________________________________________

Date: ______/______/_________

Signature of the examining dentist: __________________________________________

**APPENDIX C – HEALTH QUESTIONNAIRE**

**Date: ____/____/_______ Number of Participant:** ___________________

**Birthday date: ____/____/_______** Skin color: ______________________

**Telephone:**

**Smoker:** ( ) Yes ( ) No ( ) Ex-smoker

**MEDICAL HISTORY**

**Doctor’s name: _________________________________________________________**

**Doctor’s telephone: _____________________________________________________**

**Date of the last visit to the Doctor: _________________________________________**

**Name of an emergency contact: ___________________________________________**

**Telephone of an emergency contact: _______________________________________**

**Current condition of your systemic health is:** ( ) Good ( ) Regular ( ) Bad

**Have you ever undergone a serious surgery procedure?** ( ) Yes ( ) No

**If yes, explain: _________________________________________________**

**Are you doing any medical treatment?** ( ) Yes ( ) No

If yes, explain: __________________________________________________

**This year (2022), have you been vaccinated against common flu (Influenza)?** ( ) Yes ( ) No

Have you been vaccinated against **SARS-CoV-2 (Covid-19)?** ( ) Yes ( ) No

Have you ever been treated or diagnosed with any of these conditions?

( ) Abnormal bleeding

( ) Hemophilia

( ) Blood transfusion

( ) Ulcer/Colitis

( ) Heart problems

( ) Epilepsy/fainting

( ) Asthma

( ) Arthritis

( ) Glaucoma

( ) Drug abuse

( ) Skin problems

( ) Blood diseases

( ) Chemotherapy/Radiotherapy

( ) Diabetes/Abnormal blood sugar

( ) Breathing difficulties

( ) Lung problems

( ) High or low blood pressure

( ) Anemia

( ) AIDS

( ) Cancer/Tumor

( ) Kidney or liver disease

( ) Hepatitis/Jaundice

( ) Emphysema

( ) Rheumatic fever

Please, describe the conditions cited above: __________________________________________________________________________________________________________________________________________________________________________________________________________________

Height:______ Weight: _______ Blood pressure: _________

Are you allergic to any oral care product, personal hygiene product, or any of its ingredients? ( ) Yes ( ) No

If there is, explain: ____________________________________

Do you use any medication daily?

| **Name of the medication** | **Dose** | **Daily frequency** | **Reason** |
| --- | --- | --- | --- |
|  |  |  |  |
|  |  |  |  |
|  |  |  |  |
|  |  |  |  |
|  |  |  |  |

**DENTAL HISTORY**

**Dentist’s name: _________________________________________________________**

**Dentist’s telephone: _____________________________________________________**

**Date of the last visit to the Dentist: ________________________________________**

**What is the frequency that you visit your dentist: ____________________________**

**Telephone of an emergency contact: _______________________________________**

**JUST FOR WOMEN**

You are pregnant? ( ) Yes ( ) No If so, for how many months? _____________

Are you currently breastfeeding? ( ) Yes No

**APPENDIX D – SOFT AND HARD ORAL TISSUE EXAMINATION FORM**

**Number of participant: Date: _/ __/**________

What is the experimental period of this evaluation: ( ) Baseline ( ) 30 days ( ) 90 days

| Area | Normal | |  | |
| --- | --- | --- | --- | --- |
| 1. Soft palate | Yes ( ) | No ( ) | |  |
| 2. Hard palate | Yes ( ) | No ( ) | |  |
| 3. Gingival mucosa | Yes ( ) | No ( ) | |  |
| 4. Oral mucosa | Yes ( ) | No ( ) | |  |
| 5. Mucogingival folds | Yes ( ) | No ( ) | |  |
| 6. Tongue | Yes ( ) | No ( ) | |  |
| 7. Sublingual and submandibular areas | Yes ( ) | No ( ) | |  |
| 8. Salivary glands | Yes ( ) | No ( ) | |  |
| 9. Tonsillar and pharyngeal areas | Yes ( ) | No ( ) | |  |

If any of your responses it’s a No, explain: ____________________________________________________________________________________________________________________________________________

Date: __/__/__ Signature of the examiner dentist:

# **APPENDIX E – INITIAL EXAM FORM**

**Number of participant: ___________ Age: ________Date: ____/____/____**

1. Is the participant between 18 years old and 70 years old? ( ) Yes No

2. Is the participant available to participate in the entire study? ( ) Yes No

3. Does the participant have good systemic health? ( ) Yes No

4. Is the participant willing to provide information related to their medical history?

( ) Yes ( ) No

1. Did the participant sign the informed consent form? ( ) Yes No

**If, for questions 1 to 5, there is a “no” answer, the participant is ineligible for the study. He should be dismissed, and question 14 should be completed. If the subject is eligible, complete questions 6-13.**

6. Is the participant participating in another clinical trial or has participated in a clinical trial in the last month? ( ) Yes ( ) No

7. Is the participant pregnant or breastfeeding? ( ) Yes ( ) No

8. Does the participant have a history of allergies to oral hygiene products, personal hygiene products, or their ingredients? ( ) Yes ( ) No

9. Does the participant currently have mouth irritation or use oral anesthetics?

( ) Yes ( ) No

10. Does the participant have diabetes? ( ) Yes ( ) No

11. Is the participant immunocompromised (HIV, AIDS, immunosuppressive drug therapy)? ( ) Yes ( ) No

12. Does the participant use complete dentures? ( ) Yes ( ) No

13. Does the participant have carpal tunnel syndrome or arthritis in the hands?

( ) Yes ( ) No

**If, for questions 6 to 13, there is a “yes” answer, the participant is ineligible for the study. He should be dismissed, and question 14 should be completed.**

14. Is the subject eligible to enter the study? ( ) Yes No ( )

Date: ________ Signature of the examining dentist: _______________________

**APPENDIX F – DAILY LOG QUESTIONNAIRE**

| **Wisconsin Survey of Upper Airway Respiratory Symptoms (WURSS-21) - Symptom Diary** |
| --- |
| Day: Date: Hour: ID: |

**Fill in ONE circle for each of the following:**

|  | I don’t feel Sick  **0** | Very lightly  **1** | **2** | Lightly  **3** | **4** | Moderately  **5** | **6** | Seriously  **7** |
| --- | --- | --- | --- | --- | --- | --- | --- | --- |
| How sick do you feel today? |  |  |  |  |  |  |  |  |

**Rate the average severity of each cold symptom over the past 24 hours:**

|  | Don’t have this symptom  **0** | Very light  **1** | **2** | Light  **3** | **4** | Moderate  **5** | **6** | Serious  **7** |
| --- | --- | --- | --- | --- | --- | --- | --- | --- |
| Runny nose |  |  |  |  |  |  |  |  |
| Stuffy nose |  |  |  |  |  |  |  |  |
| Sneeze |  |  |  |  |  |  |  |  |
| Sore throat |  |  |  |  |  |  |  |  |
| Throat scratching |  |  |  |  |  |  |  |  |
| Cough |  |  |  |  |  |  |  |  |
| Hoarseness |  |  |  |  |  |  |  |  |
| Pressure in the head |  |  |  |  |  |  |  |  |
| Pressure in the chest |  |  |  |  |  |  |  |  |
| Tiredness |  |  |  |  |  |  |  |  |

If you have experienced at least ONE of the symptoms above, respond below:

- In the last 24 hours, how much did your cold interfere with your ability to:

|  | No way  **0** | Very lightly  **1** | **2** | Lightly  **3** | **4** | Moderately  **5** | **6** | Seriously  **7** |
| --- | --- | --- | --- | --- | --- | --- | --- | --- |
| Think clearly |  |  |  |  |  |  |  |  |
| Sleep well |  |  |  |  |  |  |  |  |
| Breathe easily |  |  |  |  |  |  |  |  |
| Walking, climbing stairs, exercising |  |  |  |  |  |  |  |  |
| Perform daily activities |  |  |  |  |  |  |  |  |
| Working outside home |  |  |  |  |  |  |  |  |
| Working inside home |  |  |  |  |  |  |  |  |
| Interact with people |  |  |  |  |  |  |  |  |
| Live your personal life |  |  |  |  |  |  |  |  |

- Compared to yesterday, I feel that my cold is:

| Much better | Better | A little better | Same | A little worst | Worst | Much worst |
| --- | --- | --- | --- | --- | --- | --- |
|  |  |  |  |  |  |  |

# **APPENDIX G – INSTRUCTIONS LABEL FOR PARTICIPANT**

**Control group:**

| **CRO-2022-04-FLU-REG-BZ-ZM**  **Product XXX**  *Brush your teeth’s twice a day (in the morning and at night) for two minutes*  *Do not ingest.*  *Restrict use for the participants of this research.*  *Only adults. Keep out of touch of children’s.*  *In emergency case, please contact* ***Dr. Maísa Casarin – (Telephone number omitted for privacy purposes)*** |
| --- |

**Test group:**

| **CRO-2022-04-FLU-REG-BZ-ZM**  **Product XXX**  *Brush your teeth’s twice a day (in the morning and at night) for two minutes.*  *After toothbrushing, gargle your mouth with 20ml of mouthwash for 30 seconds, twice a day.*  *Do not ingest.*  *Restrict use for the participants of this research.*  *Only adults. Keep out of touch of children’s.*  *In na emergency case, please contact:*  ***Dr. Maísa Casarin – (Telephone number omitted for privacy purposes)*** |
| --- |

# **APPENDIX H – VISIT FORM**

Participant number: Date: __/__/__

Is the patient currently taking any medication? (including over-the-counter products)

( ) Yes No ( )

In affirmative case, list all medications:

| **Medication** | **Total daily dose** | **Start date** | **End date (circle the “C” in case of a medication for continuous use** | **Indication** |
| --- | --- | --- | --- | --- |
|  |  |  | **C** |  |
|  |  |  | **C** |  |
|  |  |  | **C** |  |
|  |  |  | **C** |  |

All medications must be reviewed by the study supervisor.

1. Have there been any unexpected or serious reactions since the previous exam? ( ) Yes No ( )

2. For the condition mentioned above, was there any treatment prescribed? ( ) Yes No ( ) ; If yes, please describe: ______________________________________________________________________

3. Has any dental treatment been performed since the previous examination? ( ) Yes No ( );

If yes, please describe: ______________________________________________________________________

4. Since the last exam, have any medications been prescribed? ( ) Yes No ( );

If yes, describe dose, duration and reason: ______________________________________________________________________

5. Is the volunteer pregnant or breastfeeding? ( ) N/A ( ) Yes ( ) No

6. Do any of the answers to questions 1 to 5 justify the exclusion of the participant's data from the statistical analysis? ( ) Yes No ( );

If yes, please explain: ______________________________________________________________________

7. Will the participant continue in the study? ( ) Yes No ( )

If the answer to question 7 is “No”, complete question 8.

8. Did the participant complete the entire study? ( ) Yes No ( )

If not, please explain: ____________________________________________________________________________________________________________________________________________

Date: ______ Signature of the examining dentist: ______________________________
