# Supplementary material for: Efficacy of regular gargling with a cetylpyridinium chloride plus zinc containing mouthwash can reduce upper respiratory symptoms
Source: PLoS One. 2025 Feb 26;20(2):e0316807. doi: 10.1371/journal.pone.0316807 (PMC11864509; doi:10.1371/journal.pone.0316807)
Supplement: S3 Appendix — (PDF) [file pone.0316807.s003.pdf]

[redacted] [redacted]

Record 4 of 4

The U.S. government does not review or approve the safety and science of all studies listed on this website.

Read our full disclaimer (https://clinicaltrials.gov/about-site/disclaimer) for details.

COMPLETED

Effect of Mouthwash in Reducing the Symptoms Associated With Flu and Cold Viruses

ClinicalTrials.gov ID NCT06479226

Sponsor Colgate Palmolive

Information provided by Colgate Palmolive (Responsible Party)

Last Update Posted 2024-06-28

Study Details Tab

Study Overview

Brief Summary

The aim of this study will be to evaluate the efficacy of regular cleaning of the oropharynx (via gargling) on the incidence of symptoms associated with flu and colds. The working hypothesis is that there will be a significant reduction in symptoms associated with flu and colds after using the mouthwash product, compared to the experimental control regime. A total of 150 individuals will be randomized to one of the two experimental regimens and followed for a period of 90 days. The experimental regimen consists of toothbrushing followed by gargling with a cetylpyridinium chloride (**CPC**) (0.075%) and zinc containing alcohol free rinse and the control regimen consists of toothbrushing alone. Participants will be instructed to brush their teeth twice a day for two minutes. Those allocated to the test regime will gargle with 20 ml of the mouthwash after each brushing. At the initial visit, participants will receive the products and instructions for use, as well as a daily log questionnaire. Participants will be asked to send their daily records once a week, using a messaging application. The proportion of days without any of the evaluated symptoms will be used as the main outcome. In addition, soft and hard tissue exams will be performed at the initial consultation, after

30 and 90 days of follow-up by a blinded researcher. Potential adverse events will be collected throughout the study. The groups will be compared using the chi-square test and one-way analysis of variance (ANOVA) will be used to compare the treatment group symptom rates between groups. The significance level will be set at 95%.

Official Title

Efficacy of Cetylpyridinium Chloride and Zinc Mouthwash in Reducing the Occurrence of Symptoms Associated With the **Flu** and Cold Season - a Randomized Clinical Trial

Conditions

Healthy

Intervention / Treatment

- Drug: cetylpyridinium chloride (0.075%) and zinc alcohol free mouthwash

Other Study ID Numbers

- CRO-2022-04-FLU-REG-BZ-ZM

Study Start (Actual)

2022-06-05

Primary Completion (Actual)

2022-10-06

Study Completion (Actual)

2022-10-06

Enrollment (Actual)

150

Study Type

Interventional

Phase

Phase 3

Resource links provided by the National Library of Medicine

MedlinePlus (https://medlineplus.gov/) related topics: Flu (https://medlineplus.gov/flu.html)

Drug Information (https://dailymed.nlm.nih.gov/dailymed/) available for:
Cetylpyridinium chloride (https://dailymed.nlm.nih.gov/dailymed/search.cfm?labeltype=human&query=Cetylpyridinium+chloride) Zinc, elemental (https://dailymed.nlm.nih.gov/dailymed/search.cfm?labeltype=human&query=Zinc%252C+elemental) Chlorine (https://dailymed.nlm.nih.gov/dailymed/search.cfm?labeltype=human&query=Chlorine)

FDA Drug and Device Resources (https://clinicaltrials.gov/fda-links)

Contacts and Locations

This section provides the contact details for those conducting the study, and information on where this study is being conducted.

Brazil

Rio Grande Do Sul Locations

Pelotas, Rio Grande Do Sul, Brazil
Federal University of Pelotas

Click to view interactive map

Participation Criteria

Researchers look for people who fit a certain description, called eligibility criteria. Some examples of these criteria are a person's general health condition or prior treatments.

For general information about clinical research, read Learn About Studies (https://clinicaltrials.gov/study-basics/learn-about-studies).

Eligibility Criteria

Description

Inclusion Criteria:

- Good systemic general health as determined by study investigators; Availability of 90 days to participate in the study;

Exclusion Criteria:

1. Those participating in any other clinical study; 2. Pregnant or breastfeeding; 3. Presence a history of allergies to oral hygiene products, personal hygiene products, or their ingredients; 4. Have mouth irritation or use oral anesthetic sprays; 5. Have diabetes; 6. Be undergoing extensive dental treatment or oral surgery during the study; 7. Present immunocompromised (HIV, AIDS, immunosuppressive drug therapy); 8. Use complete dentures; 9. Do not have carpal tunnel syndrome or arthritis in the hands. 10. Participant who substantially fails to follow the required protocols; 11. Participant who fails to attend scheduled appointments; 12. Participant who is treated, during the study period, with medications that may interfere with the parameters being analyzed in the study; 13. Participant who is treated by a medical or dental service, and this may interfere with the parameters being analyzed in the study; 14. Participant who develops serious adverse reactions. 15. The participant who chooses to terminate their participation in the study; 16. Participant reports being pregnant during the study.

Ages Eligible for Study

18 Years to 70 Years (Adult, Older Adult)

Sexes Eligible for Study

All

Accepts Healthy Volunteers

Yes

Study Plan

This section provides details of the study plan, including how the study is designed and what the study is measuring.

How is the study designed?

Design Details

Primary Purpose : Prevention
Allocation : Randomized
Interventional Model : Parallel Assignment
Masking : Single (Investigator)

Arms and Interventions

Participant Group/Arm	Intervention/Treatment
No Intervention: Control<br><br>Toothbrushing alone	
Experimental: cetylpyridinium chloride (0.075%) and zinc alcohol free mouthwash<br><br>Toothbrushing + mouthrinse	Drug: cetylpyridinium chloride (0.075%) and zinc alcohol free mouthwash<br><br>• mouthrinse

What is the study measuring?

Primary Outcome Measures

Outcome Measure	Measure Description	Time Frame
Upper Respiratory Symptom	any upper respiratory system associated with colds and flu according to the Wisconsin Upper Respiratory Symptom Survey (WURSS-21) Daily Symptom Report.	90 days

Collaborators and Investigators

This is where you will find people and organizations involved with this study.

Sponsor

Colgate Palmolive

Study Record Dates

These dates track the progress of study record and summary results submissions to ClinicalTrials.gov. Study records and reported results are reviewed by the National Library of Medicine (NLM) to make sure they meet specific quality control standards before being posted on the public website.

Study Registration Dates

First Submitted

2024-06-24

First Submitted that Met QC Criteria

2024-06-24

First Posted

2024-06-28

Study Record Updates

Last Update Submitted that met QC Criteria

2024-06-24

Last Update Posted

2024-06-28

Last Verified

2024-06

More Information

Terms related to this study

Additional Relevant MeSH Terms

Anti-Infective Agents, Local
Anti-Infective Agents
Cetylpyridinium

Drug and device information, study documents, and helpful links

Studies a U.S. FDA-Regulated Drug Product

No

Studies a U.S. FDA-Regulated Device Product

No

Product Manufactured in and Exported from the U.S.

No

[redacted] [redacted]

Record 4 of 4

The U.S. government does not review or approve the safety and science of all studies listed on this website.

Read our full disclaimer (https://clinicaltrials.gov/about-site/disclaimer) for details.

COMPLETED

Effect of Mouthwash in Reducing the Symptoms Associated With Flu and Cold Viruses

ClinicalTrials.gov ID NCT06479226

Sponsor Colgate Palmolive

Information provided by Colgate Palmolive (Responsible Party)

Last Update Posted 2024-06-28

Researcher View Tab

Trial Contacts

Contacts	ICMJE
Contact information is only displayed when the study is recruiting subjects	

Study Record Dates

First Submitted	ICMJE
2024-06-24	

First Posted	ICMJE
2024-06-28	

Last Update Posted

2024-06-28

Last Verified *

2024-06

Outcome Measures

Change History

No changes posted

Primary (Current) * ICMJE

(Submitted: 2024-06-24)

- Upper Respiratory Symptom [Time Frame: 90 days]
  - any upper respiratory system associated with colds and flu according to the Wisconsin Upper Respiratory Symptom Survey (WURSS-21) Daily Symptom Report.

Primary (Original) * ICMJE

Same as current

Secondary (Current) [*] ICMJE

Not provided

Secondary (Original) [*] ICMJE

Not provided

Other Pre-specified (Current)

Not provided

Other Pre-specified (Original)

Not provided

Trial Description

Brief Title * ICMJE

Effect of Mouthwash in Reducing the Symptoms Associated With **Flu** and Cold Viruses

Official Title *§ ICMJE

Efficacy of Cetylpyridinium Chloride and Zinc Mouthwash in Reducing the Occurrence of Symptoms Associated With the **Flu** and Cold Season - a Randomized Clinical Trial

Brief Summary *

The aim of this study will be to evaluate the efficacy of regular cleaning of the oropharynx (via gargling) on the incidence of symptoms associated with flu and colds. The working hypothesis is that there will be a significant reduction in symptoms associated with flu and colds after using the mouthwash product, compared to the experimental control regime. A total of 150 individuals will be randomized to one of the two experimental regimens and followed for a period of 90 days. The experimental regimen consists of toothbrushing followed by gargling with a cetylpyridinium chloride (**CPC**) (0.075%) and zinc containing alcohol free rinse and the control regimen consists of toothbrushing alone. Participants will be instructed to brush their teeth twice a day for two minutes. Those allocated to the test regime will gargle with 20 ml of the mouthwash after each brushing. At the initial visit, participants will receive the products and instructions for use, as well as a daily log questionnaire. Participants will be asked to send their daily records once a week, using a messaging application. The proportion of days without any of the evaluated symptoms will be used as the main outcome. In addition, soft and hard tissue exams will be performed at the initial consultation, after 30 and 90 days of follow-up by a blinded researcher. Potential adverse events will be collected throughout the study. The groups will be compared using the chi-square test and one-way analysis of variance (ANOVA) will be used to compare the treatment group symptom rates between groups. The significance level will be set at 95%.

Detailed Description

Not provided

Study Type * ICMJE

Interventional

Study Phase * ICMJE

Phase 3

Study Design *§ ICMJE

Allocation
Randomized

Interventional Model
Parallel Assignment

Masking
Single (Investigator)

Primary Purpose

Prevention

Condition * ICMJE

- Healthy

Intervention * ICMJE

- Drug: cetylpyridinium chloride (0.075%) and zinc alcohol free mouthwash
  - mouthrinse

Study Arms * ICMJE

- No Intervention: Control
  - Toothbrushing alone
- Experimental: cetylpyridinium chloride (0.075%) and zinc alcohol free mouthwash
  - Toothbrushing + mouthrinse
  - Interventions:
    - Drug: cetylpyridinium chloride (0.075%) and zinc alcohol free mouthwash

Publications

(Includes general and study results' publications, and Pubmed publications referencing this study by ClinicalTrials.gov Identifier (NCT Number))

Not provided

Recruitment Information

Recruitment Status * ICMJE

Completed

Enrollment (Actual) *§ ICMJE

(Submitted: 2024-06-24)

150

Original Enrollment (Actual) *§ ICMJE

Same as current

Study Start Date (Actual) *§ ICMJE

2022-06-05

Primary Completion Date (Actual) *

2022-10-06 (Final data collection date for primary outcome measure)

Study Completion Date (Actual) *§ ICMJE

2022-10-06

Eligibility Criteria * ICMJE

Inclusion Criteria:

- Good systemic general health as determined by study investigators; Availability of 90 days to participate in the study;

Exclusion Criteria:

1. Those participating in any other clinical study; 2. Pregnant or breastfeeding; 3. Presence a history of allergies to oral hygiene products, personal hygiene products, or their ingredients; 4. Have mouth irritation or use oral anesthetic sprays; 5. Have diabetes; 6. Be undergoing extensive dental treatment or oral surgery during the study; 7. Present immunocompromised (HIV, AIDS, immunosuppressive drug therapy); 8. Use complete dentures; 9. Do not have carpal tunnel syndrome or arthritis in the hands. 10. Participant who substantially fails to follow the required protocols; 11. Participant who fails to attend scheduled appointments; 12. Participant who is treated, during the study period, with medications that may interfere with the parameters being analyzed in the study; 13. Participant who is treated by a medical or dental service, and this may interfere with the parameters being analyzed in the study; 14. Participant who develops serious adverse reactions. 15. The participant who chooses to terminate their participation in the study; 16. Participant reports being pregnant during the study.

Sex/Gender * ICMJE

Sexes Eligible for the Study:
All

Ages * ICMJE

18 Years to 70 Years (Adult, Older Adult)

Accepts Healthy Volunteers *§ ICMJE

Yes

Location Countries ICMJE

Brazil

Removed Location Countries

Administrative Information

NCT Number ICMJE

NCT06479226

Other Study ID Numbers [*] ICMJE

CRO-2022-04-FLU-REG-BZ-ZM

Has Data Monitoring Committee

Not provided

U.S. FDA-regulated Product *§

Studies a U.S. FDA-regulated Drug Product
No

Studies a U.S. FDA-regulated Device Product
No

Product manufactured in and exported from the U.S
No

IPD Sharing Statement ICMJE

Not provided

Current Responsible Party *

Colgate Palmolive

Original Responsible Party *

Same as current

Current Study Sponsor * ICMJE

Colgate Palmolive

Original Study Sponsor * ICMJE

Same as current

Collaborators ICMJE

Not provided

Investigators ICMJE

Not provided

PRS Account

Colgate Palmolive

Symbol Legend

*	Required
*§	Required if Study Start Date is on or after January 18, 2017
[*]	Conditionally required
No Symbol	Unmarked fields are optional. If no information is provided, fields will be labeled as "not provided"
ICMJE	Data element required by the International Committee of Medical Journal Editors (https://www.icmje.org/recommendations/browse/publishing-and-editorial-issues/clinical-trial-registration.html) and the World Health Organization ICTRP (https://www.who.int/clinical-trials-registry-platform)

[redacted] [redacted]

Record 4 of 4

The U.S. government does not review or approve the safety and science of all studies listed on this website.

Read our full disclaimer (https://clinicaltrials.gov/about-site/disclaimer) for details.

COMPLETED

Effect of Mouthwash in Reducing the Symptoms Associated With Flu and Cold Viruses

ClinicalTrials.gov ID NCT06479226

Sponsor Colgate Palmolive

Information provided by Colgate Palmolive (Responsible Party)

Last Update Posted 2024-06-28

No Results Posted Tab

Results Overview

No Study Results Posted on ClinicalTrials.gov for this Study

Study results have not been submitted. This may be because the study isn't done, the deadline for submitting results hasn't passed, this study isn't required to submit results, or the sponsor or investigator has requested or received a certification to delay submitting the results.

For more information:

FDAAA 801 and the Final Rule: Which trials must have results information submitted to ClinicalTrials.gov? (https://clinicaltrials.gov/policy/fdaaa-801-final-rule#trials-results-submitted)

FDAAA 801 and the Final Rule: Delayed submission of results information (https://clinicaltrials.gov/policy/fdaaa-801-final-rule#DelayedSubmission)

Recruitment Status	Actual Primary Completion Date	Actual Study Completion Date			
Recruitment Status	Completed	Actual Primary Completion Date	2022-10-06	Actual Study Completion Date	2022-10-06

More Information

Record History

[redacted]

[redacted]

Record 4 of 4

The U.S. government does not review or approve the safety and science of all studies listed on this website.

Read our full disclaimer (https://clinicaltrials.gov/about-site/disclaimer) for details.

COMPLETED

Effect of Mouthwash in Reducing the Symptoms Associated With Flu and Cold Viruses

ClinicalTrials.gov ID NCT06479226

Sponsor Colgate Palmolive

Information provided by Colgate Palmolive (Responsible Party)

Last Update Posted 2024-06-28

Record History Tab

Study Record Versions

- This table shows all the versions of this study record arranged in order by submitted date.
  - To view one version of the study record, click the submitted date.
  - To compare two versions, select them using the check boxes and click "Compare" at the bottom of the list.

	Version	Date submitted (YYYY-MM-DD)	Changes
☐	1	2024-06-24	• None (earliest version on record)

Compare
