# Supplementary material for: Efficacy of regular gargling with a cetylpyridinium chloride plus zinc containing mouthwash can reduce upper respiratory symptoms
Source: PLoS One. 2025 Feb 26;20(2):e0316807. doi: 10.1371/journal.pone.0316807 (PMC11864509; doi:10.1371/journal.pone.0316807)
Supplement: Attachment — Submitted filename: Response letter 15.8.24.docx [file pone.0316807.s006.docx]

August 15^th^, 2024

**TO:** Allyza Magadan

*PlosOne*

Dear Dr. Magadan,

The following manuscript, entitled “Regular gargling with a Cetylpyridinium Chloride plus Zinc containing mouthwash can reduce upper respiratory symptoms” was previously submitted for the **PlosOne (ID PONE-D-24-20683).** However, the manuscript was Withdrawn from the system, as we needed additional time to answer all queries raised by the editor. We would like to thank your time and forward the manuscript once again for appreciation of the Journal.

We declare that the manuscript is not being considered for publication elsewhere and the results have not been published previously. In addition, we inform that the new final version of the manuscript has been approved by all authors.

All changes were made in the main text, but not highlighted in the manuscript, as this is a new submission. However, we provide further clarification in this response letter.

Sincerely,

We've checked your submission and before we can proceed, we need you to address the following issues:

1. Please upload a copy of your trial study protocol as a supporting information file. By the study protocol, we mean the complete and detailed plan for the conduct and analysis of the trial that the ethics committee approved before the trial began. Please send this in the original language. If this is in a language other than English, please also provide a translation. Please detail any deviations from this study protocol in the Methods section of your manuscript. Your study protocol will be made available to the editors and reviewers, and will be published as supporting information with your manuscript if accepted for publication. (If you do not agree to this, we will not be able to publish your manuscript). If you have formally published a study protocol for your trial in a journal then you should cite this in your manuscript, but you still need to send us the original document.

Trial Protocol Short or a Published Paper

Could you please confirm that the clinical trial protocol you have included as Supplementary Information is the version that was submitted to and approved by your ethics committee before the trial began? By the clinical trial protocol, we mean the complete and detailed plan for the conduct and analysis of the trial. Note that we do not accept summaries of the clinical trial protocol or published reports of the protocol. Please provide the protocol in the original language. If it is in a language other than English, please also provide a translation. (We will accept a translation of the main points of the protocol.)

Your study protocol will be made available to the editors and reviewers, and will be published as supporting information with your manuscript if accepted for publication. (If you do not agree to this, we will not be able to publish your manuscript).

**Answer:** The study protocol in both Portuguese (native language), which was approved by the ethics committee prior to data collection, and English is included in the Journal’s platform. No deviation from the protocol was performed.

2. We note that you have selected “Clinical Trial” as your article type. PLOS ONE requires that all clinical trials are registered in an appropriate registry (the WHO list of approved registries is at https://www.who.int/clinical-trials-registry-platform/network/primary-registries" https://www.who.int/clinical-trials-registry-platform/network/primary-registries and more information on trial registration is at http://www.icmje.org/about-icmje/faqs/clinical-trials-registration/).

Please state the name of the registry and the registration number (e.g. ISRCTN or ClinicalTrials.gov) in the submission data and on the title page of your manuscript.

a) Please provide the complete date range for participant recruitment and follow-up in the methods section of your manuscript.

b) If you have not yet registered your trial in an appropriate registry, we now require you to do so and will need confirmation of the trial registry number before we can pass your paper to the next stage of review. Please include in the Methods section of your paper your reasons for not registering this study before enrolment of participants started. Please confirm that all related trials are registered by stating: “The authors confirm that all ongoing and related trials for this drug/intervention are registered.

**Answer:** We have provided, in the main text, the requested information. The clinical trial registration was also performed *a posteriori.* Reasons for not registering *a priori* was also provided.

3. Please upload your clinical trial registry link in information details page clinical trial section.

**Answer:** This was also provided. The Clinical Trials registration is NCT06479226.

4. Please complete all items on the Human Participants Research Checklist that are relevant for your submission, by following this link: http://journals.plos.org/plosone/s/file?id=dc11/PLOSOne_Human_Subjects_Research_Checklist.docx (Contact us at plosone@plos.org if you cannot access the document.) There may be overlap between the checklist items and other queries listed below; please address any duplicated queries both in your response email and on the checklist itself. Upload the completed Human Participants Research Checklist as file type “Other” when you re-submit your manuscript. This document is for internal journal use only and will not be published if your article is accepted. The requested information will help us to assess whether your submission complies with PLOS ONE’s policies and adheres to applicable reporting standards. Note that your manuscript may be rejected if you provide incomplete or inadequate responses to the checklist questions and that changing the

‘Section/Category’ of your article does not affect this requirement.

**Answer:** This file was included in the system.

5. Please provide additional details regarding participant consent. In the ethics statement in the Methods and online submission information, please ensure that you have specified what type you obtained (for instance, written or verbal, and if verbal, how it was documented and witnessed). If your study included minors, state whether you obtained consent from parents or guardians. If the need for consent was waived by the ethics committee, please include this information.

**Answer:** A written informed consent was obtained from the participants prior to their inclusion. This is stated by the following sentences: “All participants read and signed an informed consent form before being enrolled in the study. Therefore, verbal and written consent was obtained.”

6. Please remove your figures from within your manuscript file, leaving only the individual TIFF/EPS image files. These will be automatically included in the reviewer’s PDF.

**Answer:** Figures were removed from the main text.

Best regards,

The authors
